# Association Between Vitamin D Deficiency and Cardiovascular Disease Risk Factors in the MENA Population: A Systematic Review and Meta-Analysis

**DOI:** 10.3390/jcm15083158

**Published:** 2026-04-21

**Authors:** Shahd Bucheeri, Abdulla Mubarak, Jarrah Aldoseri, Ayah Redha, Nitya Kumar, Sara Mohamed

**Affiliations:** 1 School of Medicine, Royal College of Surgeons in Ireland, Medical University of Bahrain (RCSI-MUB), Busaiteen 15503, Bahrain; 21200429@rcsi-mub.com (A.M.); 21200572@rcsi-mub.com (J.A.); 21200695@rcsi-mub.com (A.R.); 2 Department of Medicine, Royal College of Surgeons in Ireland, Medical University of Bahrain, Busaiteen 15503, Bahrain; nkumar@rcsi-mub.com (N.K.); samohamed@rcsi-mub.com (S.M.)

**Keywords:** vitamin D deficiency, cardiovascular disease, blood pressure, cholesterol, diabetes mellitus, body mass index, risk factors, Middle East, North Africa

## Abstract

**Background**: The Middle East and North Africa (MENA) region faces a high cardiovascular disease (CVD) burden alongside endemic serum 25(OH)D (vitamin D) deficiency. This systematic review examines the relationship between vitamin D deficiency and CVD risk factors in MENA populations. **Methods**: PubMed, Cochrane Library, and Scopus were searched from inception to 18 October 2024, for observational studies in the MENA region examining vitamin D deficiency and cardiovascular risk factors in adults. Independent data extraction was conducted. Study quality was appraised using the Joanna Briggs Institute tool and the Newcastle–Ottawa Scale, with risk of bias visualized using Robvis. Weighted mean differences in cholesterol, body mass index (BMI), and HbA1c between those with and without vitamin D deficiency were computed with random-effects meta-analysis. The protocol was registered in PROSPERO (ID: CRD42025615188) and funded by the Royal College of Surgeons in Ireland—Medical University of Bahrain. **Results**: Seventeen studies from nine MENA countries were included, predominantly cross-sectional, involving community-based and disease-specific cohorts. Vitamin D deficiency was highly prevalent and consistently associated with higher adiposity and central obesity. Several studies reported significant links between deficiency and poor glycemic control, particularly in obese and prediabetic groups. Meta-analysis demonstrated significantly higher total cholesterol (MD = 0.32; 95% CI = 0.11 to 0.52, *p* < 0.001), BMI (MD = 1.81; 95% CI = 0.68 to 2.94, *p* < 0.001), and HbA1c levels (MD = 0.31; 95% CI = 0.06 to 0.57, *p* = 0.02) in vitamin D deficient individuals, with notable heterogeneity. **Conclusions**: Vitamin D deficiency is highly prevalent in the MENA region and consistently associated with adiposity-related risk factors. Despite heterogeneity, findings underscore the need for public health strategies and further research to clarify causal pathways and population-specific interventions.

## 1. Introduction

Cardiovascular disease (CVD) remains a major cause of morbidity and mortality worldwide, including in the Middle East and North Africa (MENA) region [1]. Notably, the MENA region exhibits the highest age-standardized rates of incidence and prevalence for coronary heart disease, alongside the second-highest age-standardized rate of mortality and disability-adjusted life years, as reported by the Global Burden of Disease study [2]. Moreover, the escalating burden of CVD in this region has been exacerbated by a high prevalence of associated risk factors, most commonly dyslipidemia, hypertension, and diabetes mellitus [3]. Recent studies have highlighted a concerning link between low vitamin D levels and CVD, as well as its associated risk factors [4,5,6]. Despite the region having an abundance of sunlight, which is a major source for vitamin D, deficiencies remain highly prevalent among MENA populations, particularly among women due to cultural practices that emphasize covering, darker-skinned populations with increased melanin levels that reduce vitamin D synthesis, and insufficient durations of sunlight exposure resulting from the harsh, hot climate [6,7,8].

Furthermore, while vitamin D is widely recognized for its essential role in maintaining mineral balance and bone health, its effects extend well beyond the skeletal system to encompass a variety of chronic conditions, including autoimmune disorders, cancer, diabetes, hypertension, and CVD [9]. Additionally, vitamin D receptors (VDRs) have been identified in smooth muscle cells, cardiomyocytes, and coronary arteries [10,11,12]. Activation of these receptors has been shown to provide cardioprotective effects by inhibiting the proliferation of vascular smooth muscle cells and contributing to a systemic anti-inflammatory state [13,14,15].

Given the rising incidence of CVDs in the MENA region, there exists a significant opportunity to investigate how vitamin D deficiency relates to these conditions. The findings of this systematic review will serve as a crucial step toward understanding the potential role of vitamin D supplementation as a cost-effective preventative strategy to mitigate cardiovascular disease risk in this specific demographic, guiding future research efforts. 

## 2. Materials and Methods

### 2.1. Protocol and Guidance

This systematic review adhered to the 2020 Preferred Reporting Items for Systematic Reviews and Meta-Analysis (PRISMA) guidelines [16] and the Cochrane Handbook for Systematic Reviews [17]. The protocol was registered in PROSPERO (PROSPERO ID: CRD42025615188) and approved by the Royal College of Surgeons in Ireland—Medical University of Bahrain (533/16 October 2024).

### 2.2. Search Strategy

A comprehensive search was conducted in PubMed, Cochrane Library, and Scopus from inception until 18 October 2024. PubMed’s Medical Subject Headings (MeSH) were utilized to identify relevant terms related to vitamin D deficiency, cardiovascular risk factors, and the MENA region. Boolean operators and truncation were applied to refine and expand the search strategy (Table 1). 

### 2.3. Eligibility Criteria

Studies were eligible if they (1) had a population aged 18 years or older; (2) were conducted within the MENA region; (3) examined the association between vitamin D deficiency and one or more cardiovascular risk factors (cholesterol levels, body mass index (BMI), blood sugar, and blood pressure); (4) assessed vitamin D status; (5) were observational studies (cross-sectional, case–control, or cohort study); and (6) were published in the English language.

Studies were excluded if they: (1) did not assess vitamin D status; (2) did not examine any association between vitamin D deficiency and one or more cardiovascular risk factors; (3) did not use a standard definition of vitamin D deficiency (<20 ng/mL), contrasting with the set baseline [18]; (4) administered vitamin D supplementation to the participants; (5) were randomized control trials (RCTs), case reports, letters, editorials, opinion pieces, unpublished data, conference abstracts, or dissertations; (6) were animal or in vitro studies; (7) were conducted outside the MENA region; (8) included a population younger than 18 years or pregnant women; and (9) were published in a language other than English (Figure S1). Examples of studies excluded after full-text assessment, along with reasons for exclusion, are provided in Table S1. Studies with missing data or losses in follow-up without supporting information were marked down on their study’s quality assessment. Authors of studies that did not report outcome data in an extractable form or did not report meta-analyzable outcome measures were contacted. If no response was obtained, such studies were not included in the meta-analysis but were instead synthesized descriptively. Finally, to address missing studies, contour-enhanced funnel plots were used to visually assess the presence of publication bias (Figure S3A–C).

### 2.4. Informed Consent Statement

This study is a systematic review and meta-analysis based exclusively on data from previously published studies. All included studies reported obtaining informed consent from their participants in accordance with their respective ethical standards as appropriate. It was ensured that all included studies explicitly defined a clear ethical approval from their respective institutional review boards or research ethics committees. No direct contact with participants occurred. Additionally, no new data were collected, and therefore no additional informed consent was required.

### 2.5. Vitamin D Status Definition

Vitamin D status was defined using the Endocrine Society clinical practice guidelines as deficiency (<20 ng/mL), insufficiency (20–29 ng/mL), and sufficiency (≥30 ng/mL) [18]. These thresholds reflect current evidence most consistent with those adopted across regional vitamin D studies [19,20]. 

### 2.6. Study Selection

Records were uploaded into the Covidence software (Veritas Health Innovation, Melbourne, Australia; available online: https://www.covidence.org/; accessed on 10 October 2024) with duplicates removed. Two reviewers (S.B. and A.M.) independently screened titles and abstracts against predefined criteria. Discrepancies were resolved by a third reviewer (J.A.). Full-text screening was conducted by two independent reviewers (J.A. and A.R.) with discrepancies resolved by a third reviewer (S.B.). A total of 17 studies were identified as eligible for data extraction.

### 2.7. Data Extraction

Data extraction and quality assessment were performed independently by four reviewers (S.B., A.M., J.A. and A.R.). Each article was screened by two independent reviewers. Discrepancies were resolved through consensus. Key variables included study design, demographics, vitamin D status, and biochemical measures (e.g., lipids, blood pressure, hemoglobin A1c (HbA1c)). Units were converted to SI standards [21]. In addition to outcome measures, data were extracted on study characteristics, including country, study design, population type, sample size, age, sex, vitamin D definitions, and confounding variables adjusted for in multi-variable analysis. When relevant data were missing or unclear, information was recorded as ‘not available,’ and no assumptions were made.

### 2.8. Statistical Analysis

Quantitative synthesis was undertaken for outcomes reported in at least four studies. These comprised total cholesterol (TC), BMI, and HbA1c. The effect measure used was a raw mean difference between vitamin D deficient and sufficient groups. Given this order of computation, positive values of mean difference indicated higher values of either TC, HbA1c, or BMI in the deficient group and hence, more adverse association between vitamin D deficiency and the outcome. Weighted mean difference was computed and meta-analyzed using a random effects model outlined by DerSimonian and Laird [22]. One included study reported HbA1c values on a logarithmic scale. These values were transformed back to the original percentage (%) scale prior to analysis to ensure unit consistency across studies. No additional transformation of the raw mean difference was performed. Heterogeneity was measured using I^2^ employing the method defined by Higgins and Thompson [23]. Sensitivity analyses were performed by restricting the analysis to healthy participants. Reporting bias was evaluated visually using contour-enhanced funnel plots and quantitatively using Egger’s test. Quantitative approaches to explore sources of heterogeneity were not sought on account of less than five estimates being available for a given outcome for meta-analysis. For other outcomes, including LDL cholesterol, HDL cholesterol, triglycerides, and blood pressure, quantitative meta-analysis was not performed primarily because sufficient comparable quantitative estimates were unavailable across the included studies. Several studies reported these outcomes without providing extractable statistical parameters required for pooling (e.g., means with standard deviations or comparable effect estimates). Consequently, these outcomes were synthesized narratively. All analyses were performed using STATA version 19 (StataCorp LLC, College Station, TX, USA) [24].

### 2.9. Quality Assessment

Quality assessment was independently conducted by two reviewers (S.B. and A.M.), and a third reviewer (J.A.) settled disputes. The Joanna Briggs Institute (JBI) critical appraisal tool was used to assess cross-sectional (*n* = 13/17, 76%) studies [25]. Assessment included a checklist of eight questions, whose queries must be labelled as a “yes,” “no,” “unclear,” or “not applicable.” Scores of ≥75% (6/8 or higher) were considered low risk of bias; scores between 50% and 75% (4–5) were considered moderate risk of bias; and scores < 50% (3/8 or lower) were considered high risk of bias. Moreover, the Newcastle–Ottawa Scale (NOS) was utilized for cohort and case–control studies, evaluating three key domains: selection of study groups, comparability of groups, and ascertainment of outcomes of interest [26]. For case–control studies, selection assessment entailed appropriate case definition, representativeness of cases, cohort selection, and controls; comparability assessment entailed a comparison of cases and control; and exposure assessment entailed ascertainment of exposure, a similar method of ascertainment for both cases and controls, and non-response rate. In addition to the domains, the NOS for cohort studies includes an outcome domain, appraising an article’s outcome assessment, follow-up duration, and sufficiency of follow-up. Articles with a low risk of bias had scores ranging from 7 to 9; those with moderate risk of bias included scores ranging from 4 to 6; and high risk of bias had scores of <3. Data were visualized using the Robvis tool (available online: https://www.riskofbias.info/; accessed on 4 September 2025) [27]. The GRADE framework was not applied, as the included studies evaluated heterogeneous populations, exposures, and outcome measurements, limiting the interpretability.

## 3. Results

### 3.1. Literature Search

**Figure 1:**
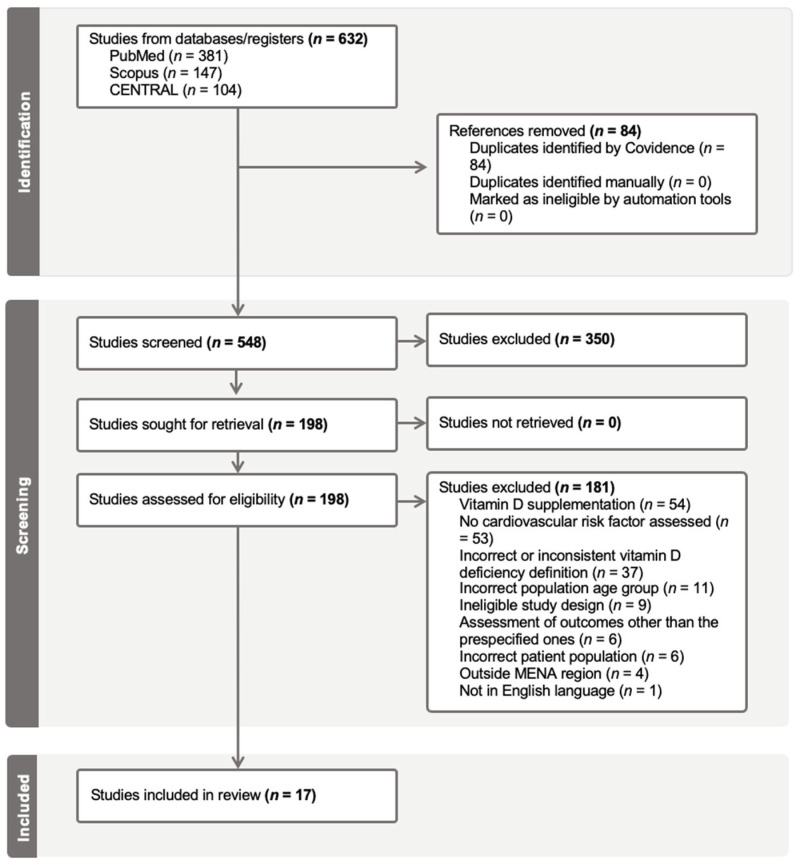
Preferred Reporting Items for Systematic Reviews and Meta-Analyses (PRISMA) flow diagram illustrating the systematic narrowing of studies from initial identification through screening and eligibility assessment to final inclusion, with exclusions documented at each stage.

The initial database search identified a total of 632 studies, of which 84 duplicates were deleted (Figure 1). Following the removal of duplicate studies, 548 articles remained for screening. Titles and abstract screenings of these articles were carefully reviewed, which was done manually by the authors (S.B. and A.M.). Thus, 350 studies were excluded, and the remaining 198 underwent full-text screening to evaluate eligibility against the redefined inclusion and exclusion criteria, resulting in 181 studies being further excluded. Reasons for exclusion included receiving vitamin D supplementation as part of the study protocol (*n* = 54), absence of assessment of any cardiovascular risk factor (*n* = 53), use of incorrect or inconsistent vitamin D deficiency definitions (*n* = 37), inclusion of non-eligible age groups (*n* = 11), ineligible study design (*n* = 9), assessment of outcomes other than the prespecified ones (*n* = 6), evaluation of the wrong patient population (*n* = 6), conduction of the study outside the MENA region (*n* = 4), and conduction of the study in a language other than English (*n* = 1). Ultimately, 17 studies met all inclusion criteria and were included for data extraction.

Studies were published between 2013 and 2024. The included studies were predominantly conducted within the Middle East (*n* = 15), spanning across nine MENA countries: Iran (*n* = 4), United Arab Emirates (*n* = 3), Saudi Arabia (*n* = 2), Bahrain (*n* = 1), Qatar (*n* = 1), Egypt (*n* = 1), Iraq (*n* = 1), Jordan (*n* = 1), and Morocco (*n* = 1). Most studies employed a cross-sectional study design (*n* = 13), followed by case–control (*n* = 2), retrospective chart review, and prospective cohort studies (*n* = 1 each). Populations varied by studies and included both community-based samples, clinic cohorts, and disease-specific groups (e.g., people with hypertension, type 2 diabetes, metabolic syndrome, and obesity). Sample sizes ranged from small single-center cohorts to larger datasets. The mean or median age reporting was inconsistent across studies, with three studies not reporting either. Likewise, study duration, when reported, was variable and ranged from 182.5 to 1188 days (*n* = 8). Table 2 summarizes the characteristics of included studies.

Definitions of vitamin D deficiency were consistent throughout studies as <20 ng/mL. Two studies further categorized these deficient patients into severe deficiency groups (<5 and <10 ng/mL). In addition, most studies (*n* = 14) reported additional definitions of vitamin D insufficiency and sufficiency. However, definitions for these variables varied by study. Most studies defined vitamin D insufficiency as 20–29 ng/mL (*n* = 6) and vitamin D sufficiency as either greater than 30 ng/mL or greater than or equal to 30 ng/mL (*n* = 10). The number of individuals with vitamin D deficiency ranged from 26 to 8889 people. 

### 3.2. Risk of Bias Assessment

**Figure 2:**
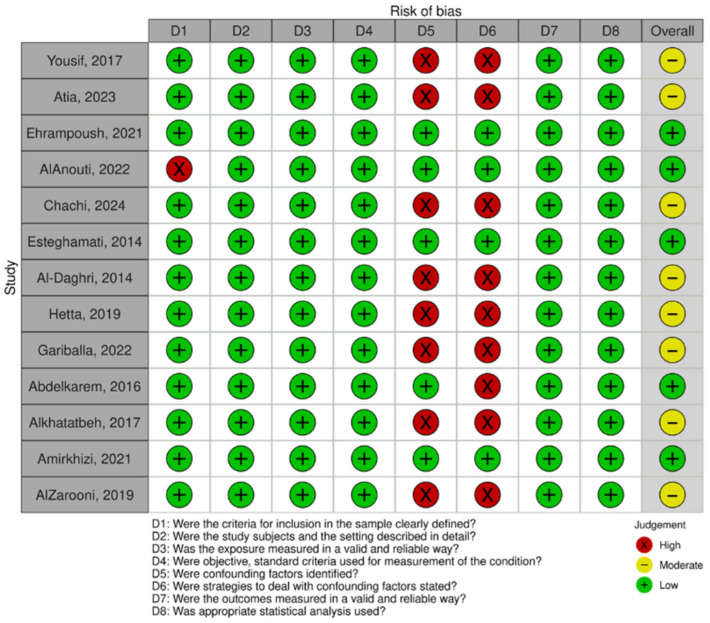
Risk of bias assessment (cross-sectional studies) using an adapted version of the Joanna Briggs Institute (JBI) Checklist for Analytical Cross-sectional Studies [25]. The Robvis tool was used for data visualization [27]. Low risk of bias is represented by JBI scores 6–8. Moderate risk of bias is represented by JBI scores 4–5. High risk of bias is represented by scores < 3. The assessment includes the following studies: [29,30,31,32,33,34,36,37,39,40,41,42,43,44].

**Figure 3:**
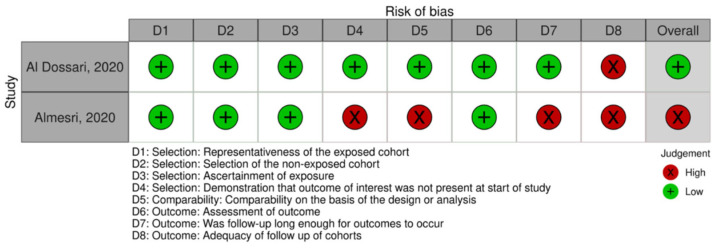
Risk of bias assessment (cohort studies) using an adapted version of the Newcastle–Ottawa Quality Assessment Scale (NOS) [26]. The Robvis tool was used for data visualization [27]. Low risk of bias is represented by NOS scores 7–9. Moderate risk of bias is represented by NOS scores 4–6. High risk of bias is represented by scores < 3. The assessment includes the following studies: [35,38].

**Figure 4:**
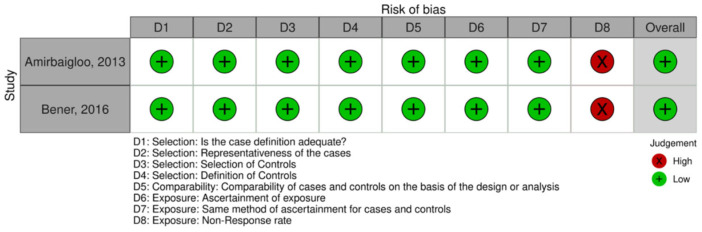
Risk of bias assessment (case–control studies) using an adapted version of the Newcastle-Ottawa Quality Assessment Scale (NOS) [26]. The Robvis tool was used for data visualization [27]. Low risk of bias is represented by NOS scores 7–9. Moderate risk of bias is represented by NOS scores 4–6. High risk of bias is represented by scores < 3. The assessment includes the following studies: [28,32].

Thirteen (76.4%) of the total seventeen studies were appraised using the JBI scale. Of those, eight (62%) studies demonstrated a moderate risk of bias, while five (38.5%) studies demonstrated a low risk of bias. High risk of bias was demonstrated across domain five (*n* = 8/13, 62%) and domain six (*n* = 9/13, 69.2%). One (*n* = 1/13, 8.0%) study scored high on the first domain. Moreover, four (*n* = 4/17, 24%) studies were appraised using the NOS. Two studies (*n* = 2/4, 50%) were appraised using the NOS cohort scale. Of those, one study (50%) demonstrated an overall low risk of bias, with one domain being scored high (domain eight). The latter study, however, demonstrated an overall high risk of bias, specifically across domains four, five, seven, and eight. Two studies (*n* = 2/4, 50%) were appraised using the NOS case–control scale. Both studies scored high on domain eight; however, they demonstrated a low overall risk of bias. Although overall study ratings were low to moderate, several studies had high risk in specific methodological domains, which may influence the reliability of pooled estimates and should be considered when interpreting the findings. The risk of bias assessment is displayed in Figure 2, Figure 3 and Figure 4.

To further assess the robustness of the findings, sensitivity analyses were conducted by excluding studies involving participants with type 2 diabetes mellitus (T2DM). The results are presented as forest plots in Figure S2, showing the pooled mean differences for TC [A], BMI [B], and HbA1c [C]. This approach allowed assessment of whether the observed associations persisted after excluding populations with a major metabolic comorbidity. Moreover, assessment of reporting bias using contour-enhanced funnel plots (Figure S3A–C) did not demonstrate visual evidence of small-study effects for TC or HbA1c, findings that were consistent with Egger’s test (*p* = 0.576 and *p* = 0.502, respectively). In contrast, the BMI analysis (Figure S3B) showed evidence of funnel plot asymmetry, with a predominance of studies reporting higher mean BMI among participants with vitamin D deficiency. Egger’s test indicated statistically significant asymmetry (*p* = 0.04), suggestive of potential small-study effects.

### 3.3. Vitamin D Deficiency and Serum Cholesterol Levels

**Figure 5:**
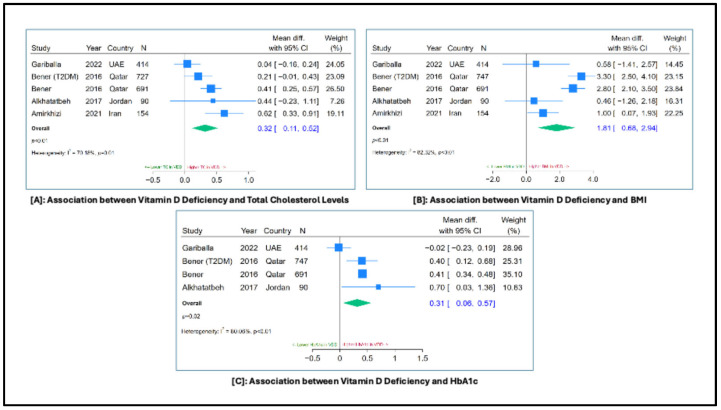
Associations of Vitamin D Deficiency with Cardiometabolic Risk Markers: Total Cholesterol, BMI, and HbA1c [32,33,39,42].

Ten studies determined serum cholesterol levels [28,29,32,33,36,37,38,39,42,43] four of which demonstrated a statistically significant inverse association between vitamin D levels and TC [33,36,38,39]. The nature of this relationship, however, varied across studies. A prospective study reported an inverse association exclusively in males, with no significant effect found in females [38]. Alkhatatbeh et al. [33], in their cross-sectional study, identified an association across all vitamin D categories—(sufficient, insufficient, and deficient)—whereas Amirkhizi et al. [39] found the association to be limited to the deficient population only. Finally, AlZarooni et al. [36] demonstrated a significant inverse relationship in the total population without stratifying participants by vitamin D status. In contrast, three studies did not report a significant association between vitamin D levels and TC levels [29,42,43], while two did not evaluate this relationship [32,37]. Quantitative synthesis (Figure 5A) indicated an inverse association between vitamin D levels, demonstrated by a positive mean difference in TC between vitamin D deficient and sufficient participants (MD = 0.32; 95% CI = 0.11 to 0.52, *p* < 0.001). However, the between-study heterogeneity was also high (I^2^ = 70%, *p* = 0.01). Restricting the analysis to healthy participants (Figure S3A) did not change the estimates (MD = 0.35; 95% CI = 0.08 to 0.63, *p* = 0.01).

### 3.4. Vitamin D Deficiency and LDL Cholesterol Levels

Nine studies measured LDL cholesterol levels, with heterogeneous findings [29,30,32,33,37,38,39,42,43]. Three studies reported a statistically significant inverse association between vitamin D deficiency and LDL levels [33,38,39]. A prospective study from Bahrain reported this correlation only among males [38]. Alkhatabeh et al. [33] reported the association in the total population, whereas Amirkhizi et al. [39] found that vitamin D deficient individuals were at higher risk of high LDL cholesterol compared to those with a sufficient vitamin D status. Conversely, three studies found no significant association [29,42,43], while the remaining three did not assess this relationship [30,32,37].

### 3.5. Vitamin D Deficiency and HDL Cholesterol Levels

Ten studies evaluated HDL cholesterol levels [28,29,30,32,33,36,38,39,42,43]. Most studies reported no significant association (*n* = 6) [30,33,37,38,39,43]. Two studies from Iran and the UAE identified a positive association between HDL cholesterol and vitamin D levels [30,36]. Esteghamati et al. [30] observed this association specifically among individuals with metabolically unhealthy obesity as defined by the International Diabetes Federation and Karelis criteria, whereas, AlZarooni et al. [36] reported the association in the overall population. The remaining two studies did not evaluate this association [28,32].

### 3.6. Vitamin D Deficiency and Triglyceride Levels

Ten studies reported triglyceride levels [28,29,30,32,33,37,38,39,42,43]. Of these, a statistically significant inverse association between vitamin D levels and triglycerides was found in two studies [29,33]. Al-Daghri et al. [29] observed this association in a healthy cohort but not among individuals with type 1 diabetes, while Alkhatatbeh et al. [33] reported the association across all vitamin D subgroups (deficient, insufficient, and sufficient). Four other studies found no significant association in the overall population [38,39,42,43], although Amirkhizi et al. [39] noted this correlation specifically within the vitamin D deficient group. The remainder of the studies did not assess this relationship [28,30,32,37].

### 3.7. Vitamin D Deficiency and Adiposity

Measures of adiposity were the most consistently reported cardiometabolic correlates of vitamin D status. Fifteen studies evaluated the BMI of the population [28,29,31,32,33,35,36,37,38,39,40,41,42,43,44], of which eight demonstrated a statistically significant inverse association between vitamin D levels and BMI [31,36,37,38,40,41,43,44]. Across studies, lower circulating vitamin D concentrations were observed among individuals with higher BMI or central adiposity, and this relationship persisted in multiple adjusted models where BMI or waist circumference remained independent correlates of vitamin D status. Six studies reported this relationship in the overall population [31,36,40,41,43,44]. One study by Hetta et al. [37] specifically observed this association among the prediabetic obese population group. Almesri et al. [38] reported this correlation exclusively in males. Two studies did not find a significant association [33,42], while five reported BMI but did not evaluate its relationship with vitamin D status [28,29,32,35,39]. Mean BMI in participants with vitamin D deficiency (Figure 5B) was found to be significantly higher as indicated by a positive mean difference in BMI between vitamin D deficient and sufficient participants (MD = 1.81; 95% CI = 0.68 to 2.94, *p* < 0.001). However, the between-study heterogeneity was also high (I^2^ = 82%, *p* < 0.01). Sensitivity analysis by excluding participants with T2DM from the analysis (Figure S3B) did not markedly change the results (MD = 1.36; 95% CI = 0.06 to 2.66, *p* = 0.04).

Similarly, waist circumference was frequently reported, with eight studies providing the mean and standard deviation values [28,29,30,33,37,39,40,44]. Among these, three showed a statistically significant inverse association between vitamin D deficiency and waist circumference in the overall population [30,40,44]. Ehrampoush et al. [40] found this association across all three stratified vitamin D status groups (mild, moderate, and severe deficiency). Two studies from Saudi Arabia and Jordan did not show a significant relationship [29,33], while the remaining three did not assess this association [28,37,39]. A minority of studies (*n* = 3) from the UAE, Morocco, and Saudi Arabia also reported body fat composition and all showed a statistically significant inverse relationship between the composition of body fat and vitamin D levels within the total population [31,41,44]. Chachi et al. [44] additionally reported this relationship within females specifically.

### 3.8. Vitamin D Deficiency and Blood Pressure

Ten studies noted the systolic and diastolic blood pressures of patients [28,30,31,32,33,34,36,37,39,42]. Four reported a statistically significant inverse relationship between systolic blood pressure (SBP) and vitamin D levels [30,33,36,39]. Esteghamati et al. [30] specifically reported this association in individuals with metabolically unhealthy obesity. In another cross-sectional study assessing both healthy cohorts and patients with hypertension, an inverse relationship with vitamin D status was observed but did not reach statistical significance [28]. Among studies that stratified their populations into the three categories of vitamin D (deficient, insufficient, and sufficient), Alkhatatbeh et al. [33] reported a significant inverse association across all categories, whereas Amirkhizi et al. [39] observed the association in the deficient population alone. Two studies did not report an association between vitamin D levels and SBP [31,42].

Of the 10 studies, only two reported a significant inverse relationship between vitamin D levels and diastolic blood pressure (DBP) [33,39]. Both also reported a significant association with SBP [33,39]. Conversely, studies showing no association with SBP likewise showed no association with DBP [31,42]. Esteghamati et al. [30] found an inverse association with SBP, but not DBP. Similarly, Alkhatatbeh et al. [33] reported an association across all three vitamin D categories for SBP but not for DBP. 

### 3.9. Vitamin D Deficiency and Glycemic Markers

Associations between vitamin D status and glycemic control were available in 14 studies [28,29,30,31,32,33,35,36,37,38,39,40,42,43]; six studies reported a statistically significant inverse association between vitamin D levels and glycemic markers [30,35,36,37,40,43], while six others reported no association [29,31,33,38,39,42]. A retrospective chart review conducted in Saudi Arabia reported a higher prevalence of vitamin D deficiency among individuals with poor glycemic control, defined as HbA1c ≥ 7%, compared with those in the controlled glycemic group [35]. Additionally, Atia et al. [40] reported a significant inverse association between vitamin D levels and both fasting blood glucose (FBG) and HbA1c levels, with vitamin D deficiency strongly associated with insulin resistance, especially in obese patients. Ehrampoush et al. [40] stratified patients into mildly, moderately, and severely deficient groups. They reported that FBG levels in moderate and severe vitamin D deficiencies were 1.04 times less than that in mild vitamin D deficiency, which was statistically significant [40]. Moreover, Esteghamati et al. [30] reported that only subjects with metabolically healthy obesity had an inverse association between vitamin D levels and FBG and HbA1c after adjusting for confounders. A cross-sectional study conducted in Egypt reported that among 101 obese prediabetic patients, a significant inverse association between vitamin D levels and FBG was found [37]. Furthermore, AlZarooni et al. [36] revealed that impaired glucose tolerance was high in participants with vitamin D deficiency. The weighted mean difference in HbA1c values between vitamin D deficient individuals and those with sufficient levels (Figure 5C) was significantly high (MD = 0.31; 95% CI = 0.06 to 0.57, *p* = 0.02), although there was considerable heterogeneity in the estimates (I^2^ = 80%, *p* < 0.01). Sensitivity analyses were conducted to see whether restricting the analysis to only healthy participants would impact the results (Figure S3C). We found that although the overall estimate did not change, the confidence intervals now crossed the null (MD = 0.30; 95% CI = −0.07 to 0.66, *p* = 0.11).

As for fasting insulin levels, six studies reported measured values [30,31,33,37,40,43]. Two of these studies reported statistically significant associations between vitamin D deficiency and fasting insulin levels. Atia et al. [43] found an inverse association in prediabetics; however, Ehrampoush et al. [40] reported a higher fasting insulin level in moderate and severe vitamin D deficiencies rather than mild deficiency. A cross-sectional study conducted in Jordan found no association between fasting insulin levels and vitamin D status [33]. The remaining three studies did not evaluate for an association between these two variables [30,31,37].

### 3.10. Geographic and Population Subgroups

Although studies varied across different geographic and climatic settings, all reported a consistently high prevalence of vitamin D deficiency, highlighting the regional burden of poor vitamin D status in countries where sunlight is abundant year-round. With respect to populations analyzed, heterogeneity was noted as well. Several studies were conducted at the level of the community, and their subjects were presumably all healthy, adult individuals [31,36,38,41,42,44]. Other studies focused on patients with cardiometabolic disorders (hypertension, type 1 or 2 diabetes, and metabolic syndrome) [28,29,30,32,33,34,35,37,39,40,43]. This permitted general population risk and disease-specific associations to be considered but introduced variability in reported outcomes as well.

Multiple studies also explored subgroup analysis based on age and sex. For instance, AlAnouti et al. [41], Chachi et al. [44], and Almesri et al. [38] highlighted differences in vitamin D status between males and females, with deficiency being more prevalent among women in two of these studies [41,44]. A study by Abdelkarem et al. [31] assessed younger age groups, while others, like Chachi et al. [44], Esteghamati et al. [30], and AlZarooni et al. [36], included middle- to older-aged populations. Collectively, these results suggest that although vitamin D deficiency is widespread in the MENA region across all categories, women and younger adults seem to be affected disproportionately, while among cardiometabolic patients, strong associations between deficiency and adverse cardiovascular risk profiles have been demonstrated. Table 3 summarizes the reported associations between vitamin D deficiency and cardiovascular risk factors.

## 4. Discussion

The most consistent relationship observed in our study was an inverse association between vitamin D deficiency and measures of adiposity, particularly BMI, waist circumference, and body fat composition. This observation is consistent with the global literature. For example, Saneei et al. [45] found a weak but significant inverse association between BMI and vitamin D levels, while Araghi et al. [46] similarly identified this association, noting that it was most prominent in individuals with an overweight or obese BMI. Yao et al. [47] describe the flow of association between vitamin D deficiency and obesity. The biological plausibility of this relationship has been described. Numerous studies explain that as a fat-soluble vitamin, vitamin D may become sequestered within adipose tissue, thereby reducing its bioavailability in the circulation [48,49,50,51]. Additionally, obesity-related metabolic disturbances may impair the conversion of vitamin D to its active form, further exacerbating deficiency in individuals with higher adiposity [52]. The stronger associations in MENA populations compared with some global cohorts could be attributed to the region’s high prevalence of obesity, variable or limited fortification of foods, and reduced outdoor activity despite abundant sunlight [53,54,55]. Together, these factors amplify vitamin D deficiency and obesity, contributing to the consistency of the observed association across the included studies.

In contrast, the relationship between vitamin D and serum lipids was less consistent. Some studies demonstrated inverse associations, while others reported no significant correlations. These findings mirror the global literature, where heterogeneity is also pronounced. For example, a cross-sectional study by Sharba et al. [56] demonstrated that vitamin D deficiency has a negative impact on the levels of cholesterol, triglycerides, HDL, and LDL. Similarly, a cross-sectional study conducted on a Chinese population showed a marked association between low vitamin D levels and elevated triglycerides and TC, particularly in females [57]. Furthermore, a meta-analysis reported that vitamin D significantly reduced triglyceride and TC levels and increased HDL levels [58]. Evidence from RCTs has likewise been mixed. Schwetz et al. [59] reported increased triglycerides, TC, LDL, and HDL levels following vitamin D supplementation. Several biological mechanisms have been proposed to explain the relationship between vitamin D status and lipid metabolism. Notably, vitamin D is thought to play a regulatory role in genes involved in cholesterol synthesis and clearance, as well as in inflammatory pathways that influence lipid metabolism [60,61,62]. Taken together, the divergence in findings suggests that low vitamin D status may serve more as a marker of poor metabolic health or increased adiposity rather than as a direct causal factor in lipid dysregulation [62].

The included studies revealed moderate evidence between vitamin D deficiency and elevated SBP, with fewer studies showing associations with DBP. This parallels global data, where inverse associations between serum vitamin D and hypertension risk were frequently documented in observational studies [63,64]. Furthermore, in a dose–response analysis, each 25 nmol/L increase in serum vitamin D level led to a 5% reduction in hypertension risk [63]. Yet, RCTs yield mixed or null results [65,66]. Moreover, most meta-analyses of RCTs have found no significant antihypertensive effect from vitamin D supplementation [67]. However, findings remain heterogeneous, with some RCTs demonstrating moderate yet statistically significant reductions in blood pressure [67]. In the MENA region, these inconsistencies may be influenced by several factors. The region exhibits a high prevalence of both hypertension and vitamin D deficiency, often coexisting within the same individual [68,69]. As deficiency is more severe and chronic in these regions than in seasonally deficient areas, observational associations may be exaggerated. Moreover, obesity, which has also been established as being highly prevalent in MENA, can independently elevate blood pressure, acting as a key confounder [70]. Several mechanisms play a role in doing so, which include stimulation of the renin–angiotensin–aldosterone system, overactivation of the sympathetic nervous system, as well as dysregulation in adipose-derived cytokines, insulin resistance, and both structural and functional changes in the kidney [71]. Some studies from the MENA region have also noted sex-specific differences, with stronger associations observed in men [72]. These differences could be due to hormonal factors or different lifestyle patterns that modulate vitamin D metabolism and blood pressure regulation [73]. Overall, while observational studies link vitamin D deficiency to hypertension, its causal role remains unclear. This uncertainty underscores the need for well-designed longitudinal and interventional studies in the MENA region, where vitamin D deficiency and cardiometabolic risk are highly prevalent [73].

In our review, associations between vitamin D status and glycemic outcomes were heterogeneous, with approximately half of the studies reporting a significant inverse association, whereas others showed no relationship. These findings align with observational studies globally, which consistently link low vitamin D to type 2 diabetes risk, insulin resistance, and impaired β-cell function [74,75]. However, global intervention studies have yielded mixed results. Large RCTs such as the D2d trial showed that vitamin D supplementation did not result in a significantly lower risk of diabetes than placebo in people at high risk for type 2 diabetes [76]. This discrepancy may be due to confounding by obesity, physical inactivity, and dietary patterns, which are highly prevalent in the MENA population [53,54,55]. Moreover, the degree of baseline deficiency in the MENA region may explain why some associations are stronger than those reported in higher-latitude countries, where deficiency is often seasonal and less severe [77].

This review presents a compelling and paradoxical public health challenge. Despite abundant sunlight, high rates of vitamin D deficiency are endemic across all nine MENA countries studied, from Saudi Arabia and Iran to Morocco. The widespread nature of this deficiency signals a regional epidemic with significant implications for cardiometabolic health. Rather than being explained by the environment alone, these patterns point to modifiable cultural, behavioural, and dietary determinants [50,51,52]. The existing literature suggests routine screening of serum vitamin D levels, integration of vitamin D fortification into national food programs, and targeted public health campaigns addressing safe sun exposure as key potential strategies [78,79]. 

Additionally, some studies have highlighted emerging analytical approaches, including artificial intelligence systems, as potential areas for future exploration in detecting vitamin D deficiency trends [80].

This review is among the first to systematically examine serum vitamin D levels in relation to a broad range of cardiovascular risk factors in the MENA region, a population often underrepresented in global analyses. The inclusion of studies with multivariable-adjusted models strengthens the validity of effect estimates, while the diversity of populations—from community-based cohorts to patients with cardiometabolic disorders—allowed us to capture both general and disease-specific associations.

### Limitations

This review has several limitations. Most included studies were cross-sectional, which limits causal inference, and leaves the direction of associations between vitamin D and cardiometabolic outcomes uncertain. The limited number of comparable effect estimates precluded meaningful subgroup, sensitivity, or meta-regression analyses, thereby restricting exploration of potential sources of heterogeneity. Additionally, the use of mean differences without confirmation of baseline comparability across study populations represents a further methodological limitation. Many studies lacked standardized effect estimates or confidence intervals, restricting comparability analysis. Small sample sizes in several studies also increased the risk of type II error. Furthermore, although the review included evidence from multiple MENA countries, geographic clustering was observed, with a predominance of studies from Iran (*n* = 4) [28,30,39,40] and Saudi Arabia (*n* = 4) [29,31,35,43], and fewer from North Africa (*n* = 2) [37,44]. This imbalance may limit the representativeness of results across the region. Therefore, additional studies from other North African countries are needed to better characterize regional variation and strengthen the overall evidence base.

These limitations were further compounded by several study-level factors influencing the strength of associations between vitamin D status and cardiometabolic risk factors, including sex differences, variation in BMI ranges, the prevalence of comorbidities, and differences in vitamin D assessment methods. Adjustment for these factors was limited in this review due to the sparse availability of individual participant data.

Finally, a degree of cultural, dietary, and environmental heterogeneity exists across countries in the MENA region. These factors include differences in dietary patterns related to local resources, physical activity behaviors, sun exposure practices, and traditional clothing, all of which can act as potential confounders [81]. While this review could not account for all these variations due to the limitations of the included studies, future longitudinal and interventional studies could explore such relationships to better clarify their effects.

## 5. Conclusions

This systematic review explored 17 studies conducted across nine countries in the MENA region, evaluating the association between vitamin D deficiency and various lipid, glycemic, and adiposity-related cardiovascular risk factors. Despite year-round sun exposure, many populations continue to exhibit vitamin D deficiency, with women and obese individuals exhibiting a higher prevalence in association. The evidence showed a consistent link between vitamin D deficiency and obesity, while findings related to lipid levels, glycemic markers, and blood pressure showed variability. Nevertheless, in the absence of RCTs, the findings of this review should be considered hypothesis-generating and not interpreted as evidence for guiding clinical or public health interventions. Future research should focus on sex- and age-specific differences in vitamin D metabolism, include underrepresented North African countries to improve regional generalizability, and prioritize longitudinal cohort studies to determine the temporality and causality of these relationships. Such efforts are crucial for developing population-specific therapies to address this deficiency epidemic.

## Figures and Tables

**Table 1:** Terms and search strategies.

PUBMED	COCHRANE LIBRARY	SCOPUS
(((((((((((((((((((((((((LDL) OR (“low density lipoprotein”)) OR (HDL)) OR (“high density lipoprotein”)) OR (BMI)) OR (“Body mass index”)) OR (triglycerides)) OR (“waist circumference”)) OR (“glomerular filtration rate”)) OR (“estimated glomerular filtration rate”)) OR (“heart disease risk factor *”)) OR (“cardiac disease risk factor *”)) OR (“cardiovascular risk factor *”)) OR (“cardiovascular disease *”)) OR (“Cholesterol, LDL” [Mesh])) OR (“Lipoproteins, LDL” [Mesh])) OR (“Lipoproteins, HDL” [Mesh])) OR (“Cholesterol, HDL” [Mesh])) OR (“Body Mass Index” [Mesh])) OR (“Triglycerides” [Mesh])) OR (“Waist Circumference” [Mesh])) OR (“Blood pressure” [Mesh])) OR (“Glucose” [Mesh])) OR (“Heart Disease Risk Factors” [Mesh])) OR (“Cardiovascular Diseases” [Mesh])) AND ((((“vitamin d deficien *”) OR (“Vitamin D Deficiency” [Mesh])) OR (((Vitamin) AND (D)) AND (deficien *)))) AND ((((((“middle east”) OR (“north africa”)) OR (“northern africa”)) OR (“Middle East” [Mesh])) OR (“Africa, Northern” [Mesh])) OR (“MENA region”))	MeSH descriptor: [Vitamin D Deficiency] explode all trees OR Vitamin D deficienc * AND “Middle East” OR MeSH descriptor: [Middle East] explode all trees OR MeSH descriptor: [Africa, Northern] explode all trees OR “MENA region” OR “North Africa” OR “Northern Africa” AND LDL OR HDL OR MeSH descriptor: [Cholesterol, LDL] explode all trees OR MeSH descriptor: [Lipoproteins, LDL] explode all trees OR MeSH descriptor: [Cholesterol, HDL] explode all trees OR MeSH descriptor: [Lipoproteins, HDL] explode all trees OR MeSH descriptor: [Triglycerides] explode all trees OR MeSH descriptor: [Body Mass Index] explode all trees OR MeSH descriptor: [Blood Pressure] explode all trees OR MeSH descriptor: [Glucose] explode all trees OR MeSH descriptor: [Waist Circumference] explode all trees OR MeSH descriptor: [Glomerular Filtration Rate] explode all trees OR triglycerides OR “body mass index” OR “blood pressure” OR glucose OR “waist circumference” OR “glomerular filtration rate” OR “estimated glomerular filtration rate” OR BMI OR eGFR OR GFR OR “cardiovascular risk factors” OR “cardiac disease risk factors” OR “heart disease risk factors” OR “cardiac disease” OR “cardiovascular disease” OR “heart disease”	(TITLE-ABS-KEY (“Vitamin D”) OR TITLE-ABS-KEY (“Calcifediol”) OR TITLE-ABS-KEY (“hydroxycholecalciferol”) AND TITLE-ABS-KEY (“Deficiency”) OR TITLE-ABS-KEY (“Insufficiency”) OR TITLE-ABS-KEY (“Deficit”) AND TITLE-ABS-KEY (“Cardiovascular” AND “Disease”) OR TITLE-ABS-KEY (“Cardiovascular” AND “risk” AND “factor”) OR TITLE-ABS-KEY (“Cardiovascular” AND “Disorder”) OR TITLE-ABS-KEY (“heart” AND “disease” AND “risk” AND “factor”) OR TITLE-ABS-KEY (“cardiovascular” AND “disease” AND “risk” AND “factor”) OR TITLE-ABS-KEY (“heart” AND “disease”) OR TITLE-ABS-KEY (“low” AND “density” AND “lipoprotein”) OR TITLE-ABS-KEY (“blood” AND “pressure”) OR TITLE-ABS-KEY (“glucose”) OR TITLE-ABS-KEY (“coronary” AND “artery” AND “disease”) OR TITLE-ABS-KEY (“hypertension”) OR TITLE-ABS-KEY (“ischemic” AND “heart” AND “disease”) OR TITLE-ABS-KEY (“estimated” AND “glomerular” AND “filtration” AND “rate”) OR TITLE-ABS-KEY (“Cholesterol”) OR TITLE-ABS-KEY (“Lipoprotein”) OR TITLE-ABS-KEY (“LDL”) OR TITLE-ABS-KEY (“HDL”) OR TITLE-ABS-KEY (“high” AND “density” AND “lipoprotein”) OR TITLE-ABS-KEY (“Triglycerides”) OR TITLE-ABS-KEY (“body” AND “mass” AND “index”) OR TITLE-ABS-KEY (“BMI”) OR TITLE-ABS-KEY (“eGFR”) OR TITLE-ABS-KEY (“glomerular” AND “filtration” AND “rate”) OR TITLE-ABS-KEY (“waist” AND “size”) OR TITLE-ABS-KEY (“Waist” AND “circumference”) AND TITLE-ABS-KEY (“middle” AND “east”) OR TITLE-ABS-KEY (“middle” AND “eastern”) OR TITLE-ABS-KEY (“mena” AND “region”) OR TITLE-ABS-KEY (“north” AND “africa”) OR TITLE-ABS-KEY (“northern” AND “africa”))

**Table 2:** Characteristics of studies assessing the association of serum vitamin D levels and cardiovascular risk factors.

Study (Author, Year)	Study Design	Study Population	Control Group	Country	Duration (Days)	Mean Age	Outcomes Assessed	Total Number of Vitamin D Deficient Cases
Amirbaigloo et al., 2013 [28]	Case–control	Patients with metabolic syndrome	Healthy controls	Iran	NA	40.8	Serum cholesterol, HDL, triglycerides, fasting glucose, BP, BMI, waist circumference	192 (59.2%, *n* = 324)
Al-Daghri et al., 2014 [29]	Cross-sectional	Type 1 diabetic patients	Healthy controls	Saudi Arabia	NA	25.9	Serum cholesterol, LDL, HDL, triglycerides, fasting glucose, BMI, waist circumference	NA
Esteghamati et al., 2014 [30]	Cross-sectional	Obese adults	NA	Iran	1188	49.58	LDL, HDL, triglycerides, fasting glucose, HbA1c, fasting insulin, BP, waist circumference	NA
Abdelkarem et al., 2016 [31]	Cross-sectional	Young obese females	NA	Saudi Arabia	242	20.7	Fasting glucose, fasting insulin, BP, BMI, body fat composition (%)	26 (17.8%, *n* = 146)
Bener et al., 2016 [32]	Case–control	Type 2 diabetic patients	Healthy controls	Qatar	577	47.7	Serum cholesterol, LDL, HDL, triglycerides, fasting glucose, HbA1c, BP, BMI	589 (52.0%, *n* = 1132)
Alkhatatbeh et al., 2017 [33]	Cross-sectional	Patients with metabolic syndrome and deficient vitamin D levels	Patients with metabolic syndrome and sufficient vitamin D levels	Jordan	213	58.6	Serum cholesterol, LDL, HDL, triglycerides, fasting glucose, HbA1c, fasting insulin, BP, BMI, waist circumference	74 (59.7%, *n* = 124)
Yousif et al., 2017 [34]	Cross-sectional	Patients with hypertension	Healthy controls	Iraq	182.5	53.7	BP, waist-to-height ratio	60 (65.9%, *n* = 91)
Al Dossari et al., 2019 [35]	Retrospective chart review	Type 2 diabetic patients with poor glycemic control	Type 2 diabetic patients with good glycemic control	Saudi Arabia	365	43.0	Fasting glucose, HbA1c, BMI, disease state	60 (47.2%, *n* = 127)
AlZarooni et al., 2019 [36]	Cross-sectional	Healthy adults	NA	UAE	397	38.5	Serum cholesterol, fasting glucose, HbA1c, BP, BMI	8889 (72.0%, *n* = 12,346)
Hetta et al., 2019 [37]	Cross-sectional	Obese, prediabetic patients	Healthy controls	Egypt	NA	34.0	Serum cholesterol, LDL, triglycerides, fasting glucose, HbA1c, fasting insulin, BP, BMI, waist circumference	33 (32.7%, *n* = 101)
Almesri et al., 2020 [38]	Prospective	Healthy adults	NA	Bahrain	365	32.7	Serum cholesterol, LDL, HDL, triglycerides, fasting glucose, HbA1c, BMI	251 (79.9%, *n* = 314)
Amirkhizi et al., 2021 [39]	Cross-sectional	Obese adults	NA	Iran	NA	34.9	Serum cholesterol, LDL, HDL, triglycerides, BP, BMI, waist circumference	NA
Ehrampoush et al., 2021 [40]	Cross-sectional	Diabetic patients	NA	Iran	275	42.5	Fasting glucose, fasting insulin, BMI, waist circumference	NA
AlAnouti et al., 2022 [41]	Cross-sectional	Healthy adults	NA	UAE	NA	NA	HbA1c, BMI, body fat composition (%)	121 (30.3%, *n* = 399)
Gariballa et al., 2022 [42]	Cross-sectional	Healthy adults	NA	UAE	NA	37.6	Serum cholesterol, LDL, HDL, triglycerides, fasting glucose, HbA1c, BP, BMI	286 (44.1%, *n* = 648)
Atia et al., 2023 [43]	Cross-sectional	Prediabetic patients	Healthy controls	Saudi Arabia	365	NA	Serum cholesterol, LDL, HDL, triglycerides, fasting glucose, HbA1c, fasting insulin, BMI	40 (38.4%, *n* = 104)
Chachi et al., 2024 [44]	Cross-sectional	Obese adults	NA	Morocco	NA	42.3	BMI, waist circumference, body fat composition (%)	928 (76.7%, *n* = 1210)

BP: Blood Pressure, both systolic and diastolic measurements (mmHg); HDL: High-density lipoprotein; LDL: Low-density lipoprotein; BMI: Body mass index; HbA1c: Hemoglobin A1c; UAE: United Arab Emirates; NA: Not available.

**Table 3:** Summary of reported associations of vitamin D deficiency with biochemical and clinical outcomes.

Study (Author, Year)	Association of Vitamin D Deficiency and Outcomes													Confounding Factors
	Serum Cholesterol	LDL	HDL	TG	Fasting Glucose	HbA1c	Fasting Insulin	SBP	DBP	BMI	WC	BF%	Disease State	
Amirbaigloo et al., 2013 [28]	-	-	-	-	-	-	-	-	-	-	-	-	-	Waist circumference, BMI, FBG, SBP, DBP, triglycerides, HDL, smoking status
Yousif et al., 2017 [34]	-	-	-	-	-	-	-	NS	-	-	-	-	-	-
Al Dossari et al., 2019 [35]	-	-	-	-	↓	↓	-	-	-	-	-	-	↓	Age, gender, BMI
Atia et al., 2023 [43]	NS	NS	NS	NS	-	-	↓	-	-	↓	-	-	-	-
Ehrampoush et al., 2021 [40]	-	-	-	-	↓	-	↓	-	-	↓	↓	-	-	Age, gender, smoking, BMI, energy intake
AlAnouti et al., 2022 [41]	-	-	-	-	-	-	-	-	-	↓	-	↓	-	Age, gender, BMI, body fat (%)
Chachi et al., 2024 [44]	-	-	-	-	-	-	-	-	-	↓	↓	↓	-	-
Esteghamati et al., 2014 [30]	-	-	↑	-	↓	-	-	↓	NS	-	↓	-	-	-
Al-Daghri et al., 2014 [29]	NS	NS	NS	↓	NS	-	-	-	-	-	NS	-	-	Age
Hetta et al., 2019 [37]	-	-	-	-	↓	↓	-	-	-	↓	-	-	-	-
Gariballa et al., 2022 [42]	NS	NS	NS	NS	NS	NS	-	-	NS	NS	-	-	-	-
Almesri et al., 2020 [38]	↓	↓	NS	NS	NS	NS	-	-	-	↓	-	-	-	-
Abdelkarem et al., 2016 [31]	-	-	-	NS	NS	NS	-	-	NS	↓	-	↓	-	Age
Bener et al., 2016 [32]	-	-	-	-	-	-	-	-	-	-	-	-	-	Age and gender
Alkhatatbeh et al., 2017 [33]	↓	↓	NS	↓	NS	↓	NS	↓	↓	NS	NS	-	-	-
Amirkhizi et al., 2021 [39]	↓	↓	NS	NS	NS	-	-	↓	↓	-	-	-	-	Age, gender, BMI, physical activity
AlZarooni et al., 2019 [36]	↓	-	↑	-	↓	↑	-	↓	-	↓	-	-	-	-

↑: Positive association; ↓: Negative association; NS: No significant association; LDL: Low-density lipoprotein cholesterol; HDL: High-density lipoprotein cholesterol; TG: Triglycerides; FBG: Fasting blood glucose; SBP: Systolic blood pressure; DBP: Diastolic blood pressure; BMI: Body mass index; WC: Waist circumference; BF%: Body fat composition (percentage). All reported associations represent statistical significance as defined by each study’s chosen thresholds and analyses.

## Data Availability

All data generated or analyzed in this study are included in this publication, available from the corresponding author upon request. The full protocol is available through the PROSPERO database. No amendments were made to the registered protocol.

## Supplementary Materials

The following supporting information can be downloaded at https://www.mdpi.com/article/10.3390/jcm15083158/s1, Figure S1: Flow Diagram of Study Inclusion and Exclusion Criteria; Figure S2: (A) Sensitivity Analysis of Total Cholesterol with T2DM subjects excluded. (B) Sensitivity Analysis of Body Mass Index (BMI) with T2DM subjects excluded. (C) Sensitivity Analysis of HbA1c with T2DM subjects excluded. Figure S3: (A) Total cholesterol publication bias assessment. (B) Body mass index (BMI) publication bias assessment. (C) HbA1c publication bias assessment. Table S1: Examples of studies excluded after full-text assessment with reasons for exclusion. References [68,82,83,84,85,86,87,88,89,90,91,92,93,94,95,96,97,98,99,100,101,102,103,104,105,106,107,108,109,110,111,112,113,114] are cited in the Supplementary Materials.

## Abbreviations

The following abbreviations are used in this manuscript:

MENA	Middle East and North Africa
CVD	Cardiovascular disease
25(OH)D	25-hydroxyvitamin D
VDRs	Vitamin D receptors
MeSH	Medical Subject Headings
BMI	Body Mass Index
JBI	Joanna Briggs Institute
NOS	Newcastle-Ottawa Scale
PRISMA	Preferred Reporting Items for Systematic Reviews and Meta-Analyses
HDL	High-density lipoprotein
LDL	Low-density lipoprotein
HbA1c	Hemoglobin A1c
UAE	United Arab Emirates
SBP	Systolic blood pressure
DBP	Diastolic blood pressure
FBG	Fasting blood glucose
TG	Triglycerides
BF	Body fat composition
NA	Not applicable
RCT	Randomized control trial

## References

[1] Di Cesare M., Perel P., Taylor S., Kabudula C., Bixby H., Gaziano T.A., McGhie D.V., Mwangi J., Pervan B., Narula J. (2024). The Heart of the World. Glob. Heart.

[2] Manla Y., Almahmeed W. (2023). The Pandemic of Coronary Heart Disease in the Middle East and North Africa: What Clinicians Need to Know. Curr. Atheroscler. Rep..

[3] Bhagavathula A.S., Shehab A., Ullah A., Rahmani J. (2021). The Burden of Cardiovascular Disease Risk Factors in the Middle East: A Systematic Review and Meta-Analysis Focusing on Primary Prevention. Curr. Vasc. Pharmacol..

[4] Lavie C.J., Lee J.H., Milani R.V. (2011). Vitamin D and cardiovascular disease will it live up to its hype?. J. Am. Coll. Cardiol..

[5] Wang L., Song Y., Manson J.E., Pilz S., März W., Michaëlsson K., Lundqvist A., Jassal S.K., Barrett-Connor E., Zhang C. (2012). Circulating 25-hydroxy-vitamin D and risk of cardiovascular disease: A meta-analysis of prospective studies. Circ. Cardiovasc. Qual. Outcomes.

[6] Mithal A., Wahl D.A., Bonjour J.P., Burckhardt P., Dawson-Hughes B., Eisman J.A., El-Hajj Fuleihan J., Josse R.G., Lips P., Morales-Torres J. (2009). Global vitamin D status and determinants of hypovitaminosis D. Osteoporos. Int..

[7] Haq A., Svobodová J., Imran S., Stanford C., Razzaque M.S. (2016). Vitamin D deficiency: A single centre analysis of patients from 136 countries. J. Steroid Biochem. Mol. Biol..

[8] El-Hajj Fuleihan G. (2009). Vitamin D Deficiency in the Middle East and its Health Consequences. Clin. Rev. Bone Miner. Metab..

[9] Norman P.E., Powell J.T. (2014). Vitamin D and Cardiovascular Disease. Circ. Res..

[10] Merke J., Milde P., Lewicka S., Hügel U., Klaus G., Mangelsdorf D.J., Haussler M.R., Rauterberg E.W., Ritz E. (1989). Identification and regulation of 1,25-dihydroxyvitamin D3 receptor activity and biosynthesis of 1,25-dihydroxyvitamin D3. Studies in cultured bovine aortic endothelial cells and human dermal capillaries. J. Clin. Investig..

[11] Schnatz P.F., Nudy M., O’Sullivan D.M., Jiang X., Cline J.M., Kaplan J.R., Clarkson T.B., Appt S.E. (2012). The quantification of vitamin D receptors in coronary arteries and their association with atherosclerosis. Maturitas.

[12] Schnatz P.F., Nudy M., O’Sullivan D.M., Jiang X., Cline J.M., Kaplan J.R., Clarkson T.B., Appt S.E. (2012). Coronary artery vitamin D receptor expression and plasma concentrations of 25-hydroxyvitamin D: Their association with atherosclerosis. Menopause.

[13] Wu-Wong J.R., Nakane M., Ma J., Ruan X., Kroeger P.E. (2006). Effects of Vitamin D analogs on gene expression profiling in human coronary artery smooth muscle cells. Atherosclerosis.

[14] Rostkowska-Nadolska B., Sliupkas-Dyrda E., Potyka J., Kusmierz D., Fraczek M., Krecicki T., Kubik P., Zatonski M., Latocha M. (2010). Vitamin D derivatives: Calcitriol and tacalcitol inhibits interleukin-6 and interleukin-8 expression in human nasal polyp fibroblast cultures. Adv. Med. Sci..

[15] Schnatz P.F., Vila-Wright S., Jiang X., Register T.C., Kaplan J.R., Clarkson T.B., Appt S.E. (2012). The association between plasma 25-hydroxyvitamin D3 concentrations, C-reactive protein levels, and coronary artery atherosclerosis in postmenopausal monkeys. Menopause.

[16] Cantorna M.T., Hayes C.E., DeLuca H.F. (1998). 1,25-Dihydroxycholecalciferol inhibits the progression of arthritis in murine models of human arthritis. J. Nutr..

[17] Page M.J., McKenzie J.E., Bossuyt P.M., Boutron I., Hoffmann T.C., Mulrow C.D., Shamseer L., Tetzlaff J.M., Akl E.A., Brennan S.E. (2021). The PRISMA 2020 statement: An updated guideline for reporting systematic reviews. BMJ.

[18] Holick M.F., Binkley N.C., Bischoff-Ferrari H.A., Gordon C.M., Hanley D.A., Heaney R.P., Murad M.H., Weaver C.M. (2011). Evaluation, treatment, and prevention of vitamin D deficiency: An Endocrine Society clinical practice guideline. J. Clin. Endocrinol. Metab..

[19] Al-Alyani H., Al-Turki H.A., Al-Essa O.N., Alani F.M., Sadat-Ali M. (2018). Vitamin D deficiency in Saudi Arabians: A reality or simply hype: A meta-analysis (2008–2015). J. Fam. Community Med..

[20] Nakhl S., Sleilaty G., El Samad S., Saliba Y., Chahine R., Farès N. (2019). Association between vitamin D deficiency and lipid and non-lipid markers of cardiovascular diseases in the middle east region. Eur. J. Clin. Nutr..

[21] (2011). Conversion Table-Conventional to SI Units. J. Feline Med. Surg..

[22] DerSimonian R., Laird N. (1986). Meta-analysis in clinical trials. Control. Clin. Trials.

[23] Higgins J.P., Thompson S.G. (2002). Quantifying heterogeneity in a meta-analysis. Stat. Med..

[24] StataCorp LLC. https://www.stata.com/.

[25] Moola S., Munn Z., Tufanaru C., Aromataris E., Sears K., Sfetc R., Currie M., Lisy K., Qureshi L., Mattis P. (2020). Chapter 7: Systematic Reviews of Etiology and Risk. JBI Manual for Evidence Synthesis.

[26] Gualdi-Russo E., Zaccagni L. (2026). The Newcastle–Ottawa Scale for Assessing the Quality of Studies in Systematic Reviews. Publications.

[27] McGuinness L.A., Higgins J.P.T. (2021). Risk-of-bias VISualization (robvis): An R package and Shiny web app for visualizing risk-of-bias assessments. Res. Synth. Methods..

[28] Amirbaigloo A., Hosseinpanah F., Sarvghadi F., Tohidi M., Eskandary P.S., Azizi F. (2013). Absence of association between vitamin D deficiency and incident metabolic syndrome: Tehran Lipid and Glucose Study. Metab. Syndr. Relat. Disord..

[29] Al-Daghri N.M., Al-Attas O.S., Alokail M.S., Alkharfy K.M., Yakout S.M., Aljohani N.J., Al Fawaz H., Al-Ajlan A.S., Sheshah E.S., Al-Yousef M. (2014). Lower vitamin D status is more common among Saudi adults with diabetes mellitus type 1 than in non-diabetics. BMC Public Health.

[30] Esteghamati A., Aryan Z., Esteghamati A., Nakhjavani M. (2014). Differences in vitamin D concentration between metabolically healthy and unhealthy obese adults: Associations with inflammatory and cardiometabolic markers in 4391 subjects. Diabetes Metab..

[31] Abdelkarem H.M., El-Sherif M.A., Gomaa S.B. (2016). Vitamin D status and insulin resistance among young obese Saudi females. Saudi Med. J..

[32] Bener A., Al-Hamaq A.O., Kurtulus E.M., Abdullatef W.K., Zirie M. (2016). The role of vitamin D, obesity and physical exercise in regulation of glycemia in Type 2 Diabetes Mellitus patients. Diabetes Metab. Syndr..

[33] Alkhatatbeh M.J., Abdul-Razzak K.K., Khasawneh L.Q., Saadeh N.A. (2017). High Prevalence of Vitamin D Deficiency and Correlation of Serum Vitamin D with Cardiovascular Risk in Patients with Metabolic Syndrome. Metab. Syndr. Relat. Disord..

[34] Yousif R.M., Mahmood M. (2017). Vitamin D is a suggested target in hypertensive Iraqi patients management. Drug Invent. Today.

[35] Al Dossari K.K., Ahmad G., Aljowair A., Alqahtani N., Shibrayn M.B., Alshathri M., Alshehri D., Akhlaq S., Bin Hejab F., Alqahtani A. (2020). Association of vitamin d with glycemic control in Saudi patients with type 2 diabetes: A retrospective chart review study in an emerging university hospital. J. Clin. Lab. Anal..

[36] Al Zarooni A.A.R., Al Marzouqi F.I., Al Darmaki S.H., Prinsloo E.A.M., Nagelkerke N. (2019). Prevalence of vitamin D deficiency and associated comorbidities among Abu Dhabi Emirates population. BMC Res. Notes.

[37] Hetta H.F., Fahmy E.M., Mohamed G.A., Gaber M.A., Elkady A., Elbadr M.M., Al-Kadmy I.M.S. (2019). Does vitamin D status correlate with insulin resistance in obese prediabetic patients? An Egyptian multicenter study. Diabetes Metab. Syndr..

[38] Almesri N., Das N.S., Ali M.E., Gumaa K., Giha H.A. (2020). Gender-Dependent Association of Vitamin D Deficiency with Obesity and Hypercholesterolemia (LDLC) in Adults. Endocr. Metab. Immune Disord. Drug Targets.

[39] Amirkhizi F., Pishdadian A., Asghari S., Hamedi-Shahraki S. (2021). Vitamin D status is favorably associated with the cardiovascular risk factors in adults with obesity. Clin. Nutr. ESPEN.

[40] Ehrampoush E., Mirzay Razzaz J., Arjmand H., Ghaemi A., Raeisi Shahraki H., Ebrahim Babaei A., Osati S., Homayounfar R. (2021). The association of vitamin D levels and insulin resistance. Clin. Nutr. ESPEN.

[41] AlAnouti F., Ahmad A.S., Wareth L.A., Dhaheri A.A., Oulhaj A., Junaibi A.A., Al Naeemi A., Al Hamiz A., Al Hosani A., Al Zaabi E. (2022). Associations between serum 25-hydroxyvitamin D, body mass index and body fat composition among Emirati population: Results from the UAE healthy future study. Front. Endocrinol..

[42] Gariballa S., Yasin J., Abluwi G., Al Essa A. (2022). Vitamin D deficiency associations with metabolic, bone turnover and adverse general health markers in community free living adults. BMC Endocr. Disord..

[43] Atia T., Abdelzaher M.H., Nassar S.A., Gafar H.H., Husseini M.A.M., Kaabi A.M.Y., Sakr H.I. (2023). Investigating the relationship between vitamin-D deficiency and glycemia status and lipid profile in nondiabetics and prediabetics in Saudi population. Medicine.

[44] Chachi E.M., Moukal A., Aghrouch M., Farouqi A.E., Kaaya A. (2024). The Status of Vitamin D in Obese Adults in Southern Morocco: A Cross-Sectional Study. Clin. Lab..

[45] Saneei P., Salehi-Abargouei A., Esmaillzadeh A. (2013). Serum 25-hydroxy vitamin D levels in relation to body mass index: A systematic review and meta-analysis. Obes. Rev..

[46] Oliai Araghi S., van Dijk S.C., Ham A.C., Brouwer-Brolsma E.M., Enneman A.W., Sohl E., Swart K.M.A., van der Zwaluw N.L., van Wijngaarden J.P., Dhonukshe-Rutten R.A.M. (2015). BMI and Body Fat Mass Is Inversely Associated with Vitamin D Levels in Older Individuals. J. Nutr. Health Aging.

[47] Yao Y., Zhu L., He L., Duan Y., Liang W., Nie Z., Jin Y., Wu X., Fang Y. (2015). A meta-analysis of the relationship between vitamin D deficiency and obesity. Int. J. Clin. Exp. Med..

[48] Holick M.F. (2007). Vitamin D deficiency. N. Engl. J. Med..

[49] Stein E.M., Strain G., Sinha N., Ortiz D., Pomp A., Dakin G., McMahon D.J., Bockman R., Silverberg S.J. (2009). Vitamin D insufficiency prior to bariatric surgery: Risk factors and a pilot treatment study. Clin. Endocrinol..

[50] Holick M.F. (2023). The One-Hundred-Year Anniversary of the Discovery of the Sunshine Vitamin D_3_: Historical, Personal Experience and Evidence-Based Perspectives. Nutrients.

[51] Censani M., Stein E.M., Shane E., Oberfield S.E., McMahon D.J., Lerner S., Fennoy I. (2013). Vitamin D Deficiency Is Prevalent in Morbidly Obese Adolescents Prior to Bariatric Surgery. ISRN Obes..

[52] Cominacini M., Fumaneri A., Ballerini L., Braggio M., Valenti M.T., Dalle Carbonare L. (2023). Unraveling the Connection: Visceral Adipose Tissue and Vitamin D Levels in Obesity. Nutrients.

[53] Abdelgadir E., Rashid F., Bashier A., Zidan M., McGowan B., Alawadi F. (2025). Prevalence of overweight and obesity in adults from the Middle East: A large-scale population-based study. Diabetes Obes. Metab..

[54] Abdul Majeed S., Said S., Hassan D.A., Sadiq F., Alhosani M., Al-Jawaldeh A., El-Obeid T., Tayyem R. (2025). Evaluating the effectiveness and risks of bread fortification programs in the middle eastern region: A comprehensive review. Front. Public Health.

[55] Musaiger A.O., Al-Kandari F.I., Al-Mannai M., Al-Faraj A.M., Bouriki F.A., Shehab F.S., Al-Dabous L.A., Al-Qalaf W.B. (2014). Perceived barriers to weight maintenance among university students in Kuwait: The role of gender and obesity. Environ. Health Prev. Med..

[56] Sharba Z.F., Shareef R.H., Abd B.A., Hameed E.N. (2021). Association between Dyslipidemia and Vitamin D Deficiency: A Cross-Sectional Study. Folia Med..

[57] Li B., Wang J., Xu J., Xie J., Liu Q., Yang C., Zhang Z. (2025). Association between dyslipidemia and vitamin D deficiency: A cross-sectional study in Chinese healthy population. Front. Endocrinol..

[58] Radkhah N., Zarezadeh M., Jamilian P., Ostadrahimi A. (2023). The Effect of Vitamin D Supplementation on Lipid Profiles: An Umbrella Review of Meta-Analyses. Adv. Nutr..

[59] Schwetz V., Scharnagl H., Trummer C., Stojakovic T., Pandis M., Grübler M.R., Verheyen N., Gaksch M., Zittermann A., Aberer F. (2018). Vitamin D supplementation and lipoprotein metabolism: A randomized controlled trial. J. Clin. Lipidol..

[60] Speeckaert M.M., Taes Y.E., De Buyzere M.L., Christophe A.B., Kaufman J.M., Delanghe J.R. (2010). Investigation of the potential association of vitamin D binding protein with lipoproteins. Ann. Clin. Biochem..

[61] Li T., Francl J.M., Boehme S., Chiang J.Y. (2013). Regulation of cholesterol and bile acid homeostasis by the cholesterol 7α-hydroxylase/steroid response element-binding protein 2/microRNA-33a axis in mice. Hepatology.

[62] Lu T.T., Repa J.J., Mangelsdorf D.J. (2001). Orphan nuclear receptors as eLiXiRs and FiXeRs of sterol metabolism. J. Biol. Chem..

[63] Mokhtari E., Hajhashemy Z., Saneei P. (2022). Serum Vitamin D Levels in Relation to Hypertension and Pre-hypertension in Adults: A Systematic Review and Dose-Response Meta-Analysis of Epidemiologic Studies. Front. Nutr..

[64] Forman J.P., Giovannucci E., Holmes M.D., Bischoff-Ferrari H.A., Tworoger S.S., Willett W.C., Curhan G.C. (2007). Plasma 25-hydroxyvitamin D levels and risk of incident hypertension. Hypertension.

[65] Zhang D., Cheng C., Wang Y., Sun H., Yu S., Xue Y., Liu Y., Li W., Li X. (2020). Effect of Vitamin D on Blood Pressure and Hypertension in the General Population: An Update Meta-Analysis of Cohort Studies and Randomized Controlled Trials. Prev. Chronic Dis..

[66] Pilz S., Gaksch M., Kienreich K., Grübler M., Verheyen N., Fahrleitner-Pammer A., Treiber G., Drechsler C., O’Hartaigh B., Obermayer-Pietsch B. (2015). Effects of Vitamin D on Blood Pressure and Cardiovascular Risk Factors. Hypertension.

[67] Theiler-Schwetz V., Trummer C., Grübler M.R., Keppel M.H., Zittermann A., Tomaschitz A., Karras S.N., März W., Pilz S., Gängler S. (2022). Effects of Vitamin D Supplementation on 24-Hour Blood Pressure in Patients with Low 25-Hydroxyvitamin D Levels: A Randomized Controlled Trial. Nutrients.

[68] Chakhtoura M., Rahme M., Chamoun N., El-Hajj Fuleihan G. (2018). Vitamin D in the Middle East and North Africa. Bone Rep..

[69] Tailakh A., Evangelista L.S., Mentes J.C., Pike N.A., Phillips L.R., Morisky D.E. (2014). Hypertension prevalence, awareness, and control in Arab countries: A systematic review. Nurs. Health Sci..

[70] Shariq O.A., McKenzie T.J. (2020). Obesity-related hypertension: A review of pathophysiology, management, and the role of metabolic surgery. Gland Surg..

[71] AlQuaiz A., Kazi A., Fouda M., Alyousefi N. (2018). Age and gender differences in the prevalence and correlates of vitamin D deficiency. Arch. Osteoporos..

[72] Wierzbicka A., Oczkowicz M. (2022). Sex differences in vitamin D metabolism, serum levels and action. Br. J. Nutr..

[73] Lips P., Eekhoff M., van Schoor N., Oosterwerff M., de Jongh R., Krul-Poel Y., Simsek S. (2017). Vitamin D and type 2 diabetes. J. Steroid Biochem. Mol. Biol..

[74] Ozcan C., Corapcıoglu D., Cerit E.T. (2023). Relationship Between Vitamin D Levels and β Cell Function and Insulin Resistance. Cureus.

[75] Chiu K.C., Chu A., Go V.L., Saad M.F. (2004). Hypovitaminosis D is associated with insulin resistance and beta cell dysfunction. Am. J. Clin. Nutr..

[76] Pittas A.G., Dawson-Hughes B., Sheehan P., Ware J.H., Knowler W.C., Aroda V.R., Brodsky I., Ceglia L., Chadha C., Chatterjee R. (2019). Vitamin D Supplementation and Prevention of Type 2 Diabetes. N. Engl. J. Med..

[77] Mendes M.M., Darling A.L., Hart K.H., Morse S., Murphy R.J., Lanham-New S.A. (2019). Impact of high latitude, urban living and ethnicity on 25-hydroxyvitamin D status: A need for multidisciplinary action?. J. Steroid Biochem. Mol. Biol..

[78] Buttriss J.L., Lanham-New S.A., Steenson S., Levy L., Swan G.E., Darling A.L., Cashman K.D., Allen R.E., Durrant L.R., Smith C.P. (2022). Implementation strategies for improving vitamin D status and increasing vitamin D intake in the UK: Current controversies and future perspectives: Proceedings of the 2nd Rank Prize Funds Forum on vitamin D. Br. J. Nutr..

[79] Hayes A., Cashman K.D. (2017). Food-based solutions for vitamin D deficiency: Putting policy into practice and the key role for research. Proc. Nutr. Soc..

[80] Alzahrani A., Asghar M.Z. (2025). Enhancing the prediction of vitamin D deficiency levels using an integrated approach of deep learning and evolutionary computing. PeerJ Comput. Sci..

[81] Golzarand M., Mirmiran P., Jessri M., Toolabi K., Mojarrad M., Azizi F. (2012). Dietary trends in the Middle East and North Africa: An ecological study (1961 to 2007). Public Health Nutr..

[82] Abdollahi A., Kamali Sarvestani H., Rafat Z., Ghaderkhani S., Mahmoudi-Aliabadi M., Jafarzadeh B., Mehrtash V. (2021). The association between the level of serum 25(OH) vitamin D, obesity, and underlying diseases with the risk of developing COVID-19 infection: A case–control study of hospitalized patients in Tehran, Iran. J. Med. Virol..

[83] Abdulrahman M.A., Alkass S.Y., Mohammed N.I. (2022). Total and free vitamin D status among apparently healthy adults living in Duhok Governorate. Sci. Rep..

[84] Afarideh M., Ghajar A., Noshad S., Saadat M., Khajeh E., Esteghamati A. (2017). Serum 25-hydroxyvitamin D, non-alcoholic fatty liver disease and type 2 diabetes. Nutr. Metab. Cardiovasc. Dis..

[85] Ahmed A.S., Alotaibi W.S., Aldubayan M.A., Alhowail A.H., Al-Najjar A.H., Chigurupati S., Elgharabawy R.M. (2021). Factors Affecting the Incidence, Progression, and Severity of COVID-19 in Type 1 Diabetes Mellitus. Biomed Res. Int..

[86] Akdam H., Alp A. (2017). Arterial stiffness and 25-hydroxyvitamin D levels in chronic kidney disease patients. Rev. Assoc. Med. Bras..

[87] Al-Ajlan A., Krishnaswamy S., Alokail M.S., Aljohani N.J., Al-Serehi A., Sheshah E., Alshingetti N.M., Fouda M., Turkistani I.Z., Al-Daghri N.M. (2015). Vitamin D deficiency and dyslipidemia in early pregnancy. BMC Pregnancy Childbirth.

[88] Albaik M., Khan J.A., Sindi I., Akesson K.E., McGuigan F.E.A. (2022). Bone mass in Saudi women aged 20–40 years: The association with obesity and vitamin D deficiency. Arch. Osteoporos..

[89] Al-Dabhani K., Tsilidis K.K., Murphy N., Ward H.A., Elliott P., Riboli E., Gunter M., Tzoulaki I. (2017). Prevalence of vitamin D deficiency and association with metabolic syndrome in a Qatari population. Nutr. Diabetes..

[90] Al-Daghri N.M., Abd-Alrahman S.H., Panigrahy A., Al-Saleh Y., Aljohani N., Al-Attas O.S., Khattak M.N.K., Alokail M. (2018). Efficacy of Vitamin D interventional strategies in saudi children and adults. J. Steroid Biochem. Mol. Biol..

[91] Bachir Cherif A., Temmar M., Bennouar S., Bouamra A., Taleb A., Bouraghda A., Bouafia M.T. (2018). Effect of vitamin D on the variability of blood pressure in premenopausal and menopausal hypertensive women in the area of Blida (Algeria). Ann. Cardiol. Angéiol.

[92] Belen E., Sungur A., Sungur M.A. (2016). Vitamin D levels predict hospitalization and mortality in patients with heart failure. Scand. Cardiovasc. J..

[93] Buckley A.J., Barakat M.T., Holick M.F., Lessan N. (2019). Parameters of Bone and Cardiovascular Health Related to 25-Hydroxyvitamin D Status in Emirati Nationals attending Primary Care and Diabetes services: A retrospective cohort study. Sci. Rep..

[94] Chijioke O.H., Ehienagudia A.M., Akinwande O.M. (2020). Low Vitamin D Levels and Correlates Amongst Adult Nigerians in North Central Nigeria. West Afr. J. Med..

[95] Eghbali B.B., Ramezani S., Alavi C.E., Ghayeghran A.R., Herfeh S.S., Atefi A., Limouei S.R., Ansar M.M. (2022). The association of 25 (OH) D3 serum level with ischemic cerebrovascular accident risk, severity and outcome in Iranian population. Am. J. Hum. Biol..

[96] Farhat K.H., Arafa M.A., Rabah D.M., Amin H.S., Ibrahim N.K. (2019). Vitamin D status and its correlates in Saudi male population. BMC Public Health.

[97] Gariballa S., Yasin J., Alessa A. (2022). A randomized, double-blind, placebo-controlled trial of vitamin D supplementation with or without calcium in community-dwelling vitamin D deficient subjects. BMC Musculoskelet. Disord..

[98] Hashemipour S., Larijani B., Adibi H., Javadi E., Sedaghat M., Pajouhi M., Soltani A., Shafaei A.R., Hamidi Z., Fard A.R.K. (2004). Vitamin D deficiency and causative factors in the population of Tehran. BMC Public Health.

[99] Jawad I., Baee H., Ismail I. (2020). Prevalence and Correlates of Vitamin D Inadequacy in a Sample of Iraqi People. Indian J. Forensic Med. Toxicol..

[100] Kambal N., Abdelwahab S., Albasheer O., Taha S., Abdelrahman N., Bani I. (2023). Vitamin D knowledge, awareness and practices of female students in the Southwest of Saudi Arabia: A cross-sectional study. Medicine.

[101] Khashayar P., Meybodi H.R., Soltani A., Taheri E., Homami M.R., Heshmat R. (2014). Association between vitamin D levels and BMI values in an Iranian population. Clin. Lab..

[102] Li X., Liu Y., Zheng Y., Wang P., Zhang Y. (2018). The Effect of Vitamin D Supplementation on Glycemic Control in Type 2 Diabetes Patients: A Systematic Review and Meta-Analysis. Nutrients.

[103] Lotfi-Dizaji L., Mahboob S., Aliashrafi S., Vaghef-Mehrabany E., Ebrahimi-Mameghani M., Morovati A. (2019). Effect of vitamin D supplementation along with weight loss diet on meta-inflammation and fat mass in obese subjects with vitamin D deficiency: A double-blind placebo-controlled randomized clinical trial. Clin. Endocrinol..

[104] Mahmoudi L., Asadi S., Al-Mousavi Z., Niknam R. (2021). A randomized controlled clinical trial comparing calcitriol versus cholecalciferol supplementation to reduce insulin resistance in patients with non-alcoholic fatty liver disease. Clin. Nutr..

[105] Nejabat A., Emamat H., Afrashteh S., Jamshidi A., Jamali Z., Farhadi A., Talkhabi Z., Nabipour I., Larijani B., Spitz J. (2024). Association of serum 25-hydroxy vitamin D status with cardiometabolic risk factors and total and regional obesity in southern Iran: Evidence from the PoCOsteo study. Sci. Rep..

[106] Nikooyeh B., Neyestani T.R., Zahedirad M., Mohammadi M., Hosseini S.H., Abdollahi Z., Salehi F., Razaz J.M., Shariatzadeh N., Kalayi A. (2016). Vitamin D-Fortified Bread Is as Effective as Supplement in Improving Vitamin D Status: A Randomized Clinical Trial. J. Clin. Endocrinol. Metab..

[107] Ponirakis G., Elhadd T., Al Ozairi E., Brema I., Chinnaiyan S., Taghadom E., Al Kandari J., Al Wotayan R., Al Ozairi A., Aljohani N. (2022). Prevalence and risk factors for diabetic peripheral neuropathy, neuropathic pain and foot ulceration in the Arabian Gulf region. J. Diabetes Investig..

[108] Razzaghi R., Pourbagheri H., Momen-Heravi M., Bahmani F., Shadi J., Soleimani Z., Asemi Z. (2017). The effects of vitamin D supplementation on wound healing and metabolic status in patients with diabetic foot ulcer: A randomized, double-blind, placebo-controlled trial. J. Diabetes Complic..

[109] Salari A., Mahdavi-Roshan M., Hasandokht T., Gholipour M., Soltanipour S., Nagshbandi M., Javadzadeh A. (2017). Nutritional intake, depressive symptoms and vitamin D status in hypertensive patients in the north of Iran: A case–control study. Hipertens. Riesgo Vasc..

[110] Sheikh V., Mozaianimonfared A., Gharakhani M., Poorolajal J. (2020). Effect of vitamin D supplementation versus placebo on essential hypertension in patients with vitamin D deficiency: A double-blind randomized clinical trial. J. Clin. Hypertens..

[111] Vidovic N., Faid F., Pantovic A., Nikolic M., Debeljak-Martacic J., Zekovic M. (2019). Vitamin D and cardio-metabolic biomarkers: Small-scale comparative study between Libyan migrants and resident women in Serbia. Libyan J. Med..

[112] Yakout S.M., Abdi S., Alaskar A.H., Khattak M.N., Al-Masri A.A., Al-Daghri N.M. (2023). Impact of Vitamin D Status Correction on Serum Lipid Profile, Carboxypeptidase N and Nitric Oxide Levels in Saudi Adults. Int. J. Mol. Sci..

[113] Younis M.Y.G. (2024). Prevalence of vitamin D deficiency in Libya and its relation to other health disorders. Metab. Target Organ Damage.

[114] Ziaee A., Javadi A., Javadi M., Zohal M., Afaghi A. (2012). Nutritional status assessment of Minodar residence in Qazvin city, Iran: Vitamin D deficiency in sunshine country, a public health issue. Glob. J. Health Sci..

