# Supplementary material for: Association Between Vitamin D Deficiency and Cardiovascular Disease Risk Factors in the MENA Population: A Systematic Review and Meta-Analysis
Source: J Clin Med. 2026 Apr 21;15(8):3158. doi: 10.3390/jcm15083158 (PMC13118101; doi:10.3390/jcm15083158)
Supplement: Supplementary file 1 [file jcm-15-03158-s001.zip › Table S1. Examples of studies excluded after full-text assessment with reasons for exclusion.pdf]

Table S1. Examples of studies excluded after full-text assessment with reasons for exclusion

Study Author	Study Year	Country	Primary reason for exclusion
Abdollahi et al. [82]	2021	Iran	No cardiovascular risk factor assessed
Abdulrahman et al. [83]	2022	Iraq	Incorrect population age group
Afarideh et al. [84]	2017	Iran	Incorrect vitamin D deficiency definition
Ahmed et al. [85]	2021	Qatar	Use of vitamin D supplementation
Akdam et al. [86]	2017	Turkey	Incorrect vitamin D deficiency definition
Al-Ajlan et al. [87]	2015	Saudi Arabia	Incorrect patient population
Albaik et al. [88]	2022	Saudi Arabia	Use of vitamin D supplementation
Al-Dabhani et al. [89]	2017	Qatar	Use of vitamin D supplementation
Al-Daghri et al. [90]	2018	Saudi Arabia	Incorrect patient population
Bachir Cherif et al. [91]	2018	Algeria	Use of vitamin D supplementation
Belen et al. [92]	2016	Turkey	Incorrect vitamin D deficiency definition
Buckley et al. [93]	2019	United Arab Emirates	Use of vitamin D supplementation
Chakhtoura et al. [68]	2018	Lebanon	No cardiovascular risk factor assessed
Chijioke et al. [94]	2020	Nigeria	Conducted outside the MENA region
Eghbali et al. [95]	2022	Iran	No cardiovascular risk factor assessed
Farhat et al. [96]	2019	Saudi Arabia	Incorrect vitamin D deficiency definition
Gariballa et al. [97]	2022	United Arab Emirates	Incorrect study design
Hashemipour et al. [98]	2004	Iran	No cardiovascular risk factor assessed
Jawad et al. [99]	2020	Iraq	Incorrect population age group
Kambal et al. [100]	2023	Saudi Arabia	No cardiovascular risk factor assessed
Khashayar et al. [101]	2014	Iran	Incorrect vitamin D deficiency definition
Li et al. [102]	2018	China	Conducted outside the MENA region
Lofti-Dizaji et al. [103]	2019	Iran	Incorrect study design
Mahmoudi et al. [104]	2021	Iran	Incorrect study design

Nejabat et al. [105]	2024	Iran	Use of vitamin D supplementation
Nikooyeh et al. [106]	2016	Iran	Incorrect study design
Ponirakis et al. [107]	2022	Qatar	No cardiovascular risk factor assessed
Razzaghi et al. [108]	2017	Iran	Incorrect study design
Salari et al. [109]	2017	Iran	Use of vitamin D supplementation
Sheikh et al. [110]	2020	Iran	Incorrect study design
Vidovic et al. [111]	2019	Serbia	Conducted outside the MENA region
Yakout et al. [112]	2023	Saudi Arabia	Use of vitamin D supplementation
Younis et al. [113]	2024	Libya	Incorrect vitamin D deficiency definition
Ziaee et al. [114]	2012	Iran	No cardiovascular risk factor assessed
