# Supplementary figures and images for: Association Between Vitamin D Deficiency and Cardiovascular Disease Risk Factors in the MENA Population: A Systematic Review and Meta-Analysis
Source: J Clin Med. 2026 Apr 21;15(8):3158. doi: 10.3390/jcm15083158 (PMC13118101; doi:10.3390/jcm15083158)

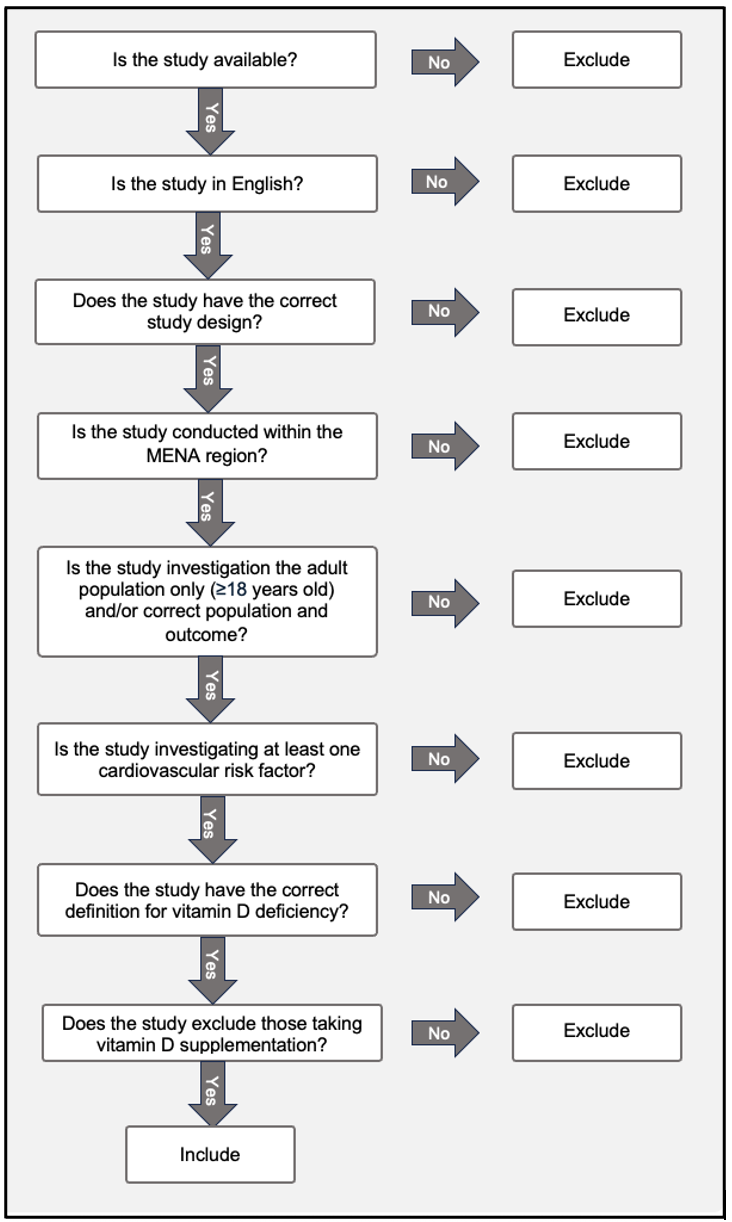

Supplement: Supplementary file 1 [file jcm-15-03158-s001.zip › Figure S1. Flow Diagram of Study Inclusion and Exclusion Criteria.png]

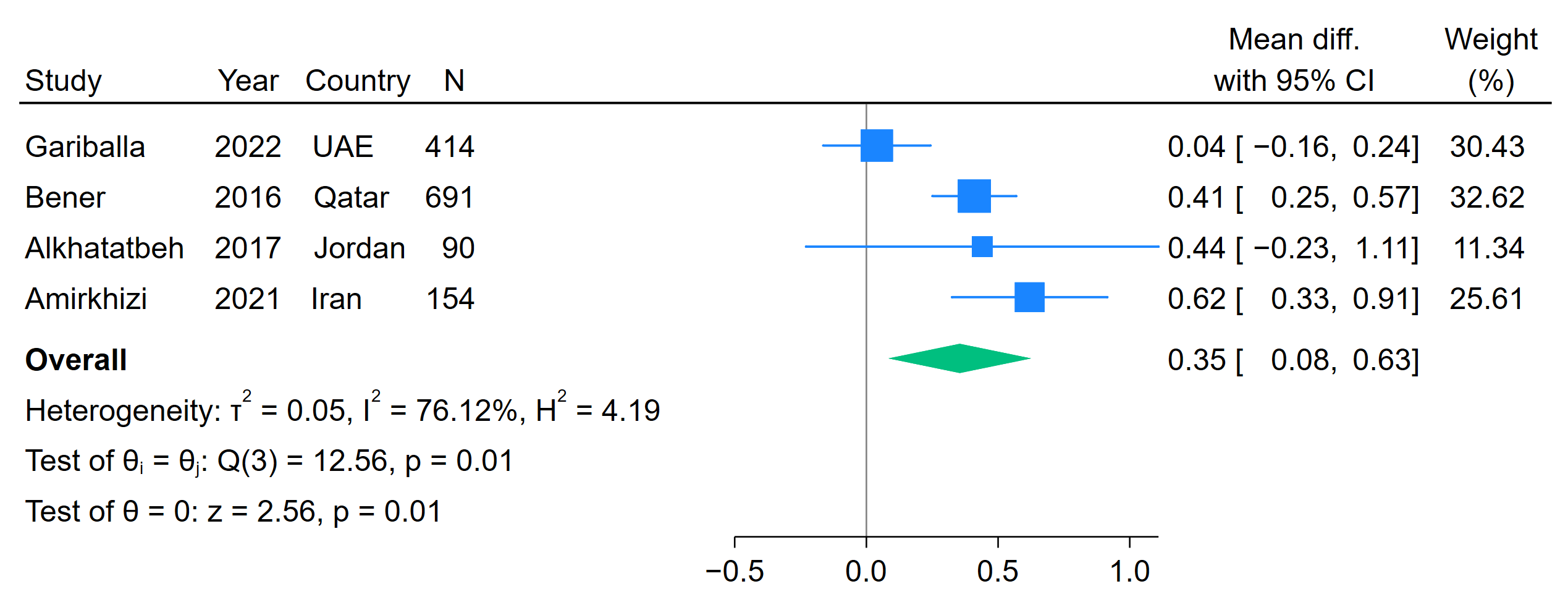

Supplement: Supplementary file 1 [file jcm-15-03158-s001.zip › Figure S2(A). Sensitivity Analysis of Total Cholesterol with T2DM subjects excluded.png]

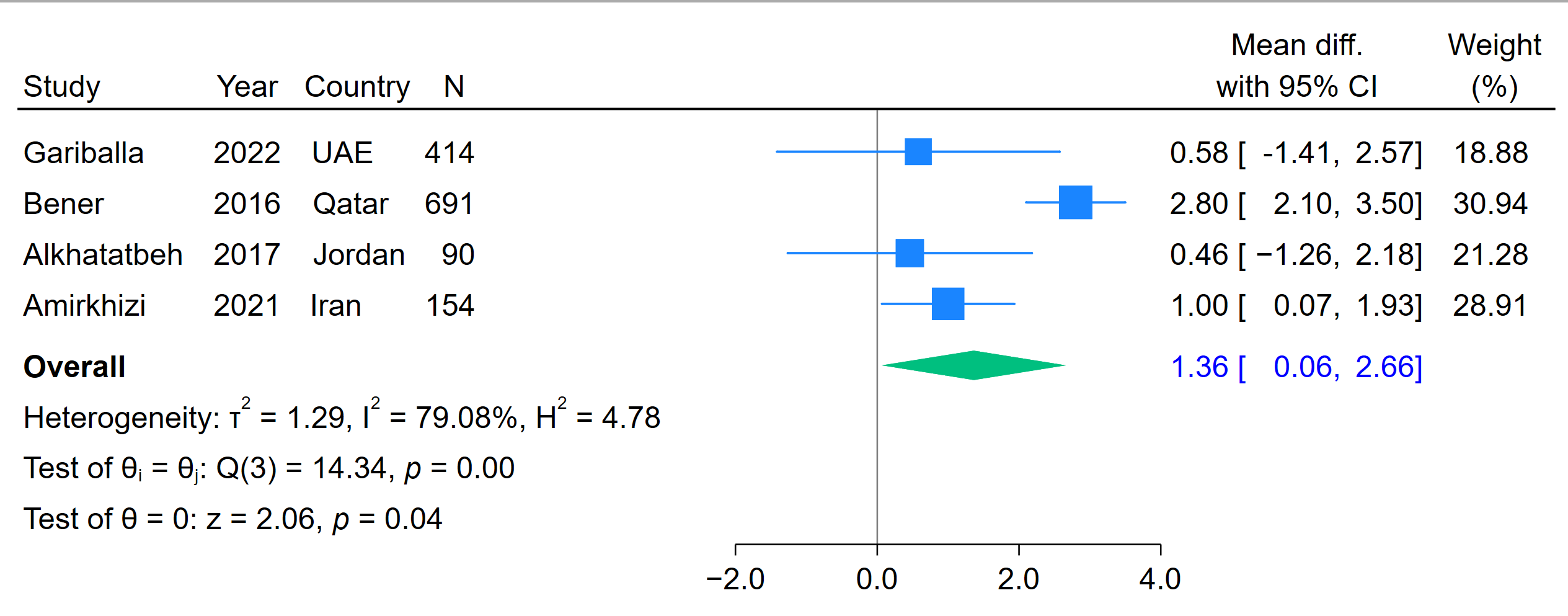

Supplement: Supplementary file 1 [file jcm-15-03158-s001.zip › Figure S2(B). Sensitivity Analysis of Body Mass Index (BMI) with T2DM subjects excluded.png]

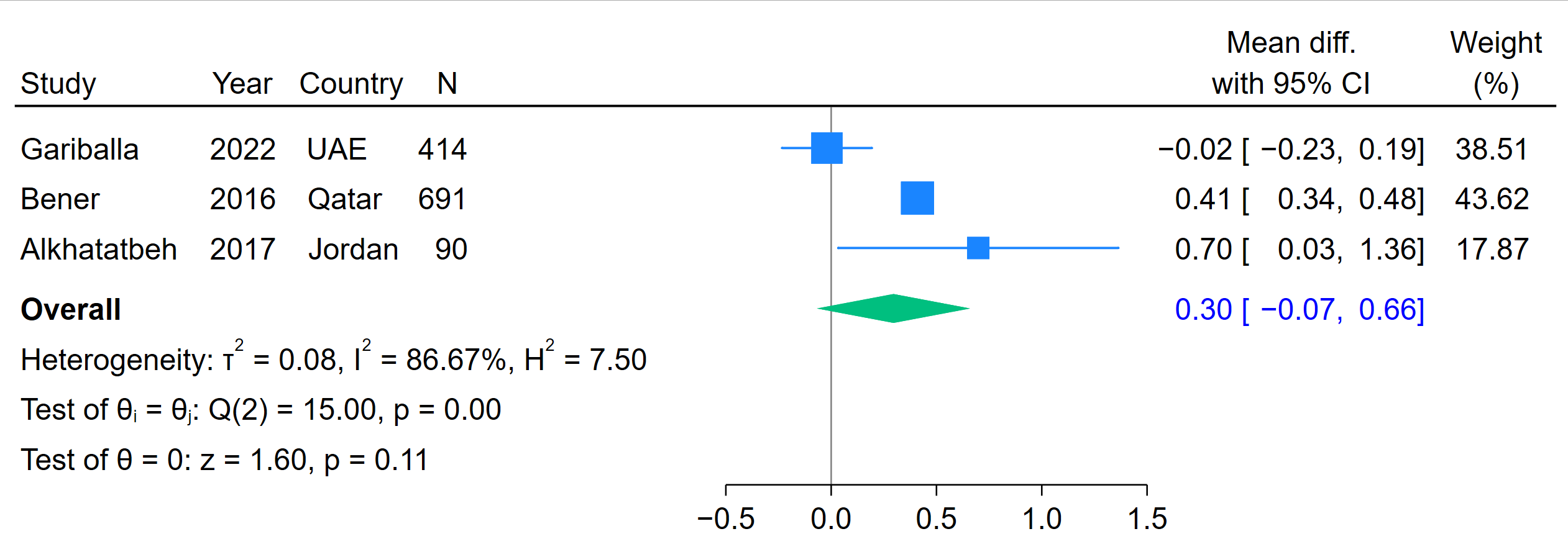

Supplement: Supplementary file 1 [file jcm-15-03158-s001.zip › Figure S2(C). Sensitivity Analysis of HbA1c with T2DM subjects excluded.png]

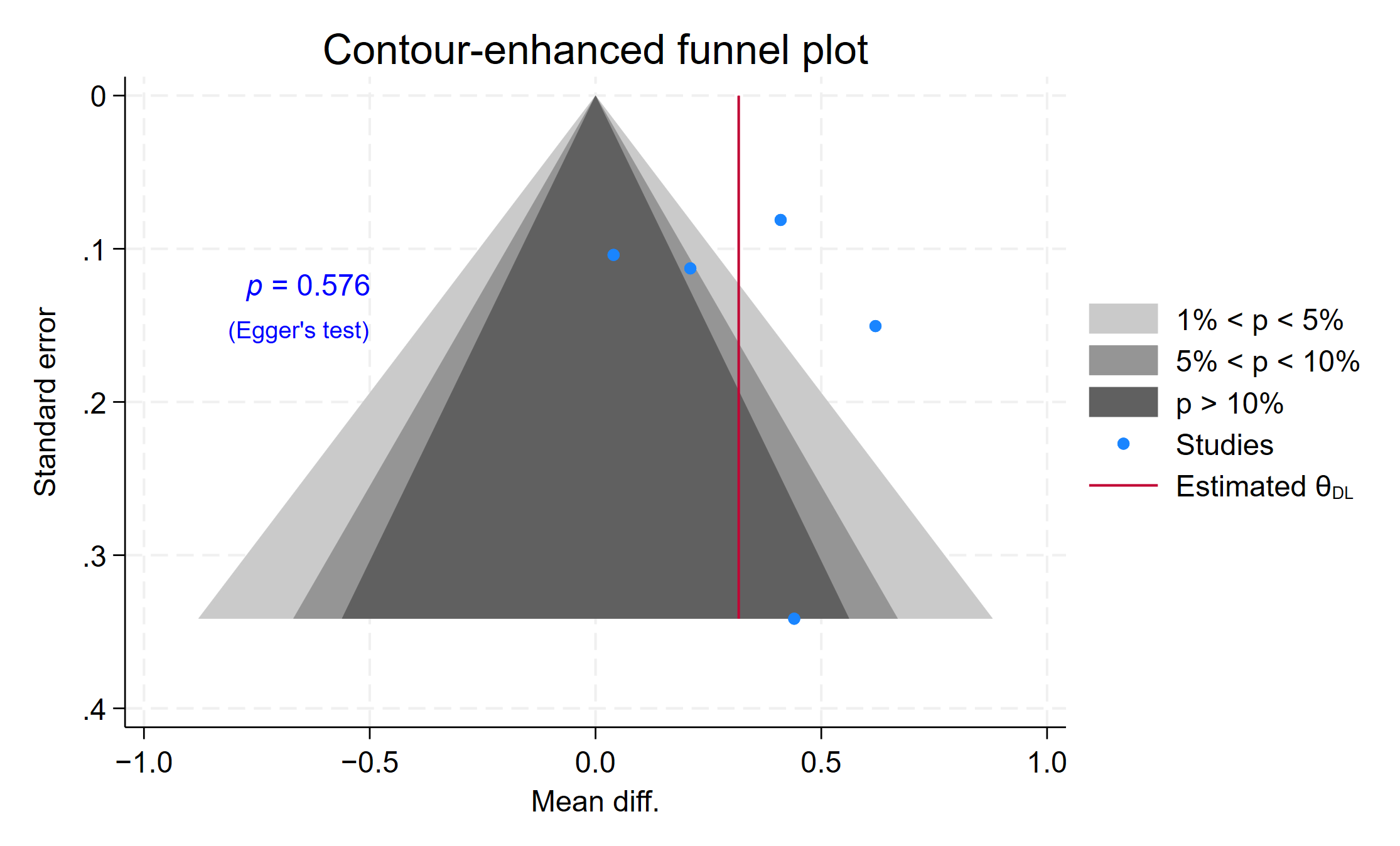

Supplement: Supplementary file 1 [file jcm-15-03158-s001.zip › Figure S3(A). Total cholesterol publication bias assessment.png]

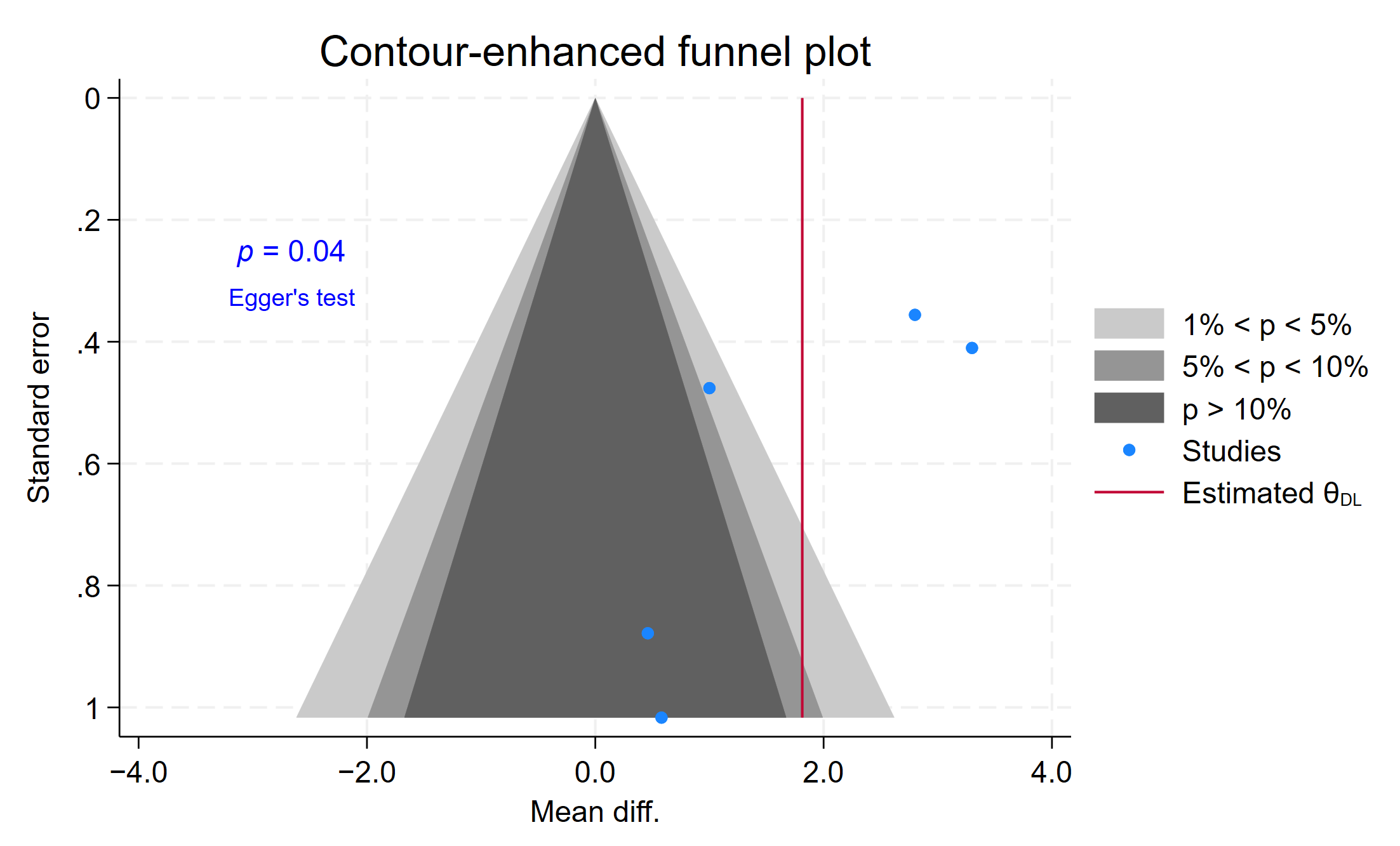

Supplement: Supplementary file 1 [file jcm-15-03158-s001.zip › Figure S3(B). Body mass index (BMI) publication bias assessment.png]

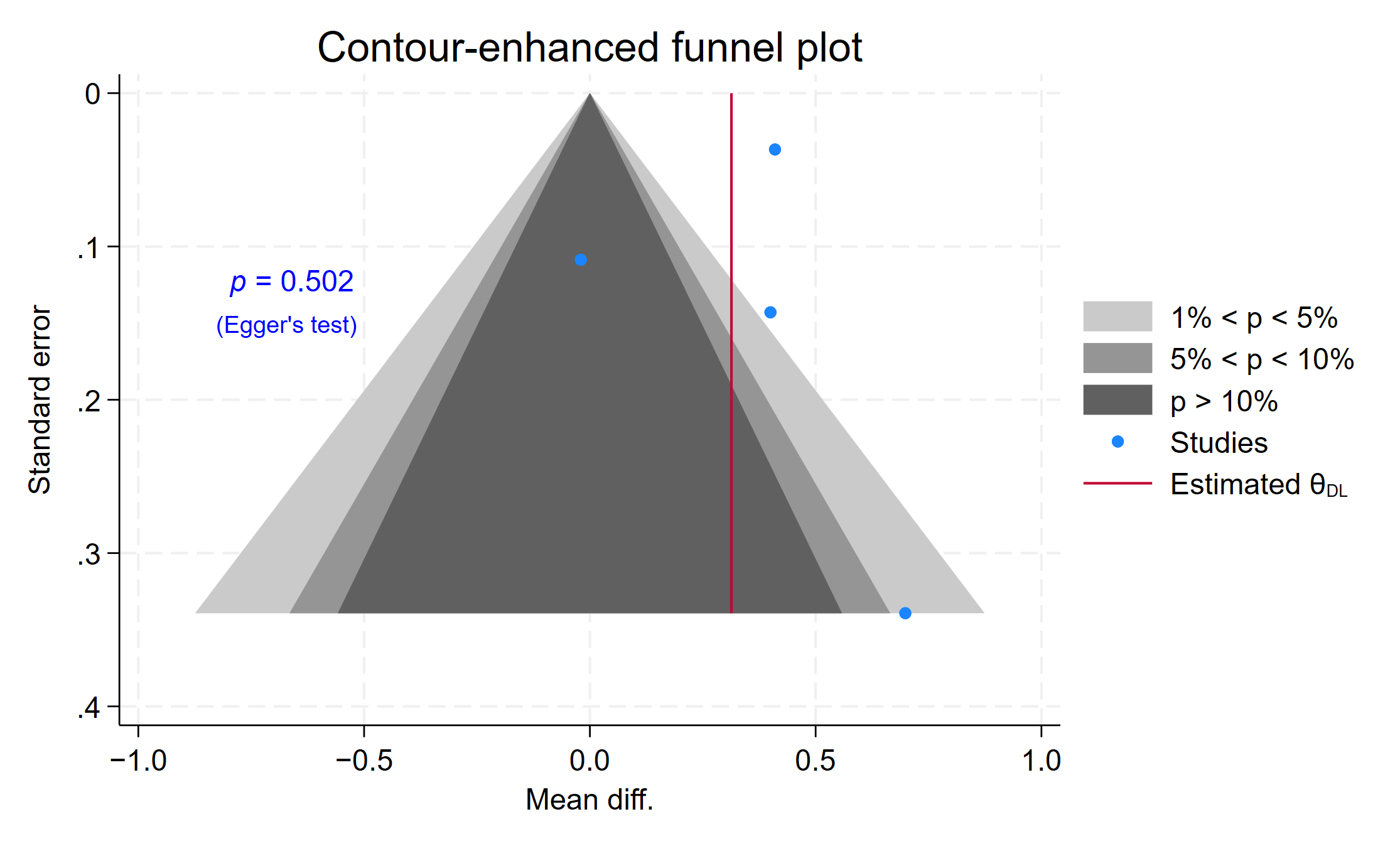

Supplement: Supplementary file 1 [file jcm-15-03158-s001.zip › Figure S3(C). HbA1c publication bias assessment.png]
